# Suppression of the postprandial hyperglycemia in patients with type 2 diabetes by a raw medicinal herb powder is weakened when consumed in ordinary hard gelatin capsules: A randomized crossover clinical trial

**DOI:** 10.1371/journal.pone.0311501

**Published:** 2024-10-09

**Authors:** Fernanda Duarte Moreira, Caio Eduardo Gonçalves Reis, Andrea Donatti Gallassi, Daniel Carneiro Moreira, Alexis Fonseca Welker

**Affiliations:** 1 Ministério da Saúde, Brasília, Brazil; 2 Secretaria de Estado de Saúde do Distrito Federal, Brasília, Brazil; 3 Programa de Pós-Graduação em Ciências e Tecnologias em Saúde, Universidade de Brasília, Brasília, Brazil; 4 Departamento de Nutrição, Universidade de Brasília, Brasília, Brazil; 5 Faculdade de Medicina, Universidade de Brasília, Brasília, Brazil; Centre Hospitalier Sud Francilien, FRANCE

## Abstract

**Introduction:**

Contradictory claims about the efficacy of several medicinal plants to promote glycemic control in patients with type 2 diabetes mellitus (T2DM) have been explained by divergences in the administration form and by extrapolation of data obtained from healthy individuals. It is not known whether the antidiabetic effects of traditional herbal medicines are influenced by gelatin capsules. This randomized crossover trial aimed to evaluate the acute effect of a single dose of raw cinnamon consumed orally either dissolved in water as a beverage or as ordinary hard gelatin capsules on postprandial hyperglycemia (>140 mg/dL; >7.8 mmol/L) in T2DM patients elicited by a nutritionally-balanced meal providing 50 g of complex carbohydrates.

**Methods:**

Fasting T2DM patients (n = 19) randomly ingested a standardized meal in five experimental sessions, one alone (Control) and the other after prior intake of 3 or 6 g of crude cinnamon in the form of hard gelatin capsules or powder dissolved in water. Blood glucose was measured at fasting and at 0.25, 0.5, 0.75, 1, 1.5 and 2 hours postprandially. After each breakfast, its palatability scores for visual appeal, smell and pleasantness of taste were assessed, as well as the taste intensity sweetness, saltiness, bitterness, sourness and creaminess.

**Results:**

The intake of raw cinnamon dissolved in water, independently of the dose, decreased the meal-induced large glucose spike (peak-rise of +87 mg/dL and Δ1-hour glycemia of +79 mg/dL) and the hyperglycemic blood glucose peak. When cinnamon was taken as capsules, these anti-hyperglycemic effects were lost or significantly diminished. Raw cinnamon intake did not change time-to-peak or the 2-h post-meal glycaemia, but flattened the glycemic curve (lower iAUC) without changing the shape that is typical of T2DM patients.

**Conclusions:**

This cinnamon’s antihyperglycemic action confirms its acarbose-like property to inhibit the activities of the carbohydrate-digesting enzymes α-amylases/α-glucosidases, which is in accordance with its exceptionally high content of raw insoluble fiber. The efficacy of using raw cinnamon as a diabetes treatment strategy seems to require its intake at a specific time before/concomitantly the main hyperglycemic daily meals. Trial registration: Registro Brasileiro de Ensaios Clínicos (ReBEC), number RBR-98tx28b.

## Introduction

Several researchers have been investigating the antidiabetic properties of different medicinal plants through diverse approaches [1, 2]. However, the statements in the conclusions of the studies are frequently contradictory. While some have concluded that a herb lowers fasting blood glucose [3–12] and HbA1c level [13–16], others came to divergent conclusions [17–26].

These contradictions are normally associated with misinterpretations of experimental data [27–29], such as the simple extrapolation to the humans based on data from animals taking large doses of the compound [1, 30, 31] or from in vitro assays [29, 31–34]. In humans, the effect of a plant is known to be changed by its administration form [1, 22, 35–40]. For example, medicinal plants are commonly consumed through swallowing hard gelatin capsules [41–47]; however, this formulation may decrease the bioavailability of bioactive molecules in relation to beverage forms [48–54]. Spices and herbs are often consumed inside a meal that undergo heating; however, some of their properties become decreased in relation to their native/raw state [55–57] and toxins/carcinogens may be formed [58–67]. Moreover, the effect of a natural product may be also different between healthy humans and patients with type 2 diabetes mellitus (T2DM) [68]. The post-meal glycemic curve of these patients has an extremely particular shape in relation to healthy people, namely a much larger post-meal blood glucose rise, higher peak levels and abnormal elevated values of 2-h post-meal blood glucose level [69–71]. Considering that abrupt high glucose ‘spikes’ are more deleterious than steady high levels of blood glucose [72–74] and that larger values of 2-hour post-meal blood glucose are associated with more severe diabetic complications [69, 75–77], evaluations of the antidiabetic potential of herbs may lead to imprecise conclusions if the particularities of the T2DM patients are not taken into account [30, 78, 79]. Some of the debatable claims about therapeutic benefits of natural products are attributed to the poor justification for experiments, i.e., not based on published papers and all available relevant data of the literature [29, 41, 80, 81].

To our knowledge, there is no study that investigated the influence of the common hard gelatin capsules on the effects of medicinal plants with antidiabetic properties. Among herbal remedies known as antidiabetic, cinnamon is one of the safest [82–85], a highly available and worldwide consumed spice [86–91], is used in Ayurvedic [79, 92–94], Traditional Chinese [1, 95, 96] and Japanese (Kampo) Medicine [1, 97–100], and is the most investigated in glucose tolerance studies [101–103]. Moreover, no study assessed its influence on the postprandial hyperglycemia (>140 mg/dL; >7.8 mmol/L) in T2DM patients elicited by a real-life common meal [104, 105]. Therefore, the aim of the present study was to investigate the acute effect of a single dose of raw cinnamon powder consumed orally either dissolved in water as a beverage or as ordinary hard gelatin capsules on postprandial hyperglycaemia in patients with T2DM elicited by a balanced breakfast providing complex carbohydrates.

## Methods

### Study design

**Fig 1:**
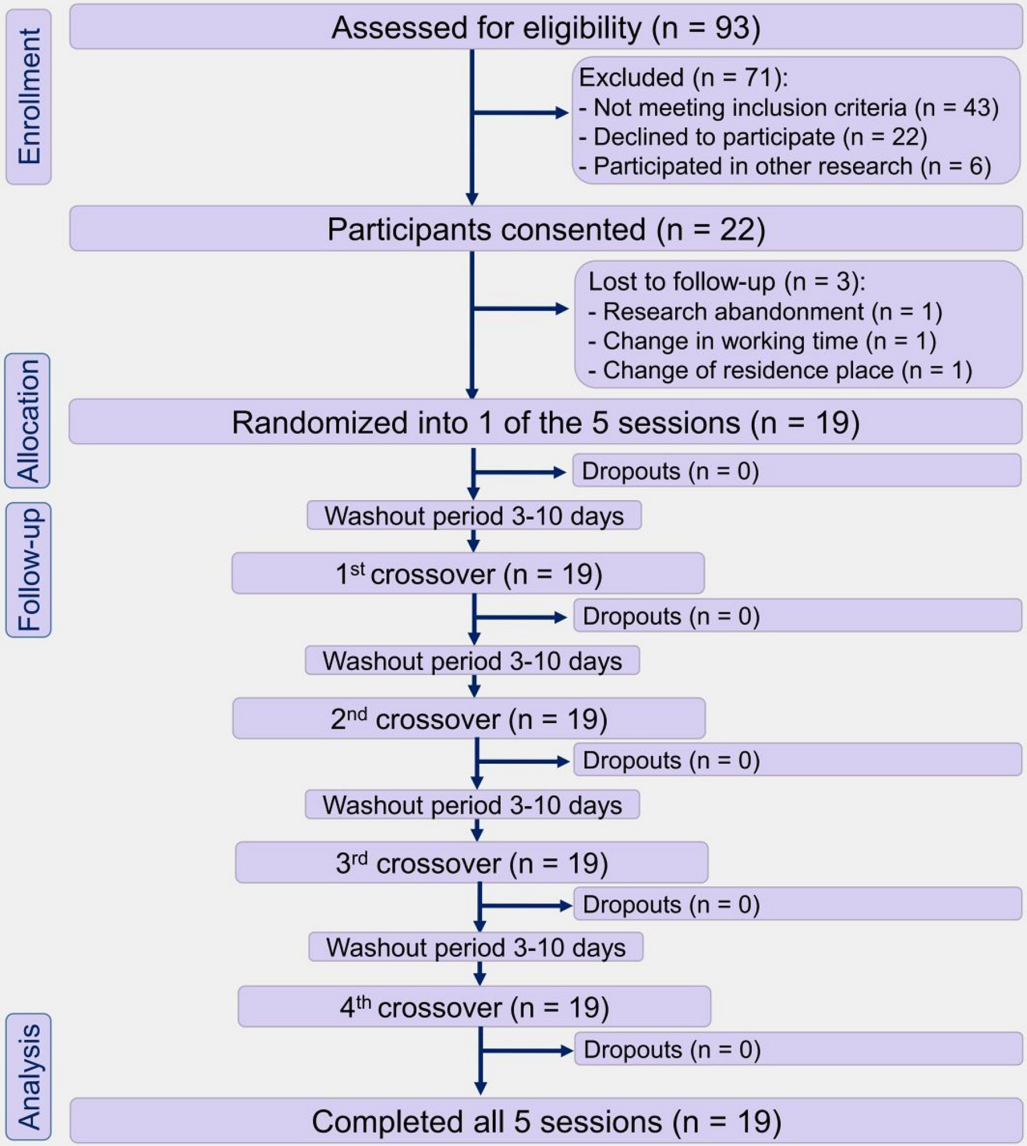
Study flowchart diagram of the participants (following CONSORT). T2DM men after eating a standardized meal alone (Control) or after prior ingestion of 3 g of raw cinnamon in capsules (3gCaps), 6 g of raw cinnamon in capsules (6gCaps), 3 g of raw cinnamon powder dissolved in water (3gPowder), or 6 g of raw cinnamon powder (6gPowder).

In this randomized crossover clinical trial, participants ingested a standardized meal on five separate test days. During the test day, each patient ingested the meal either alone (Control) or after prior intake of 3 or 6 g of raw cinnamon in the form of hard gelatin capsules or in the form of powder dissolved in water. After a washout period, each participant underwent another experimental session. The sequence in which these five procedures were administered to each participant was defined in random order (simple randomization through Excel spreadsheet). The primary outcome was postprandial blood glucose concentrations. Secondary outcomes were palatability markers. The study flow diagram is shown in Fig 1.

The protocol was approved by the Research Ethics Committee of the Foundation for Teaching and Research in Health Sciences (FEPECS/SES, for its abbreviation in Portuguese) on 4th June 2019 (decision number 3367200). The prior approval of this project (doctoral research) was a requirement of our PhD program to start the recruitment of volunteers, which occurred between 11 July 2019 and 15 October 2019. The approved trial protocol may be accessed as supplementary material in the supporting information section (S1 and S2 Protocols). Then, we conducted the study according the Declaration of Helsinki and in line with the CONSORT (Consolidated Standards of Reporting Trials) (S1 Checklist and S1 Fig). All participants were informed about the objectives of the study and provided written informed consent. Follow-up was conducted between 22 July 2019 and 28 October 2019. Although the requirements neither of the Ethics Committee nor of the PhD program did not include the registration of the protocol, this clinical trial was retrospectively registered on the publicly accessible registry https://ensaiosclinicos.gov.br on 12 November 2021 (ReBEC: RBR-98tx28b). Registration was not performed in advance because the authors thought the ethical clearance was sufficient. The authors confirm that all ongoing and related trials for this intervention are registered.

### Participants

Volunteers were recruited by advertisements using posters in public health centers in Brasília (Brazil). Eligibility criteria were: male patients with T2DM, 30–60 years of age, consistent breakfast consumption (ingestion of ≥100 kcal within 2 hours after awakening on ≥4 days per week), willingness to eat all meals, absence of allergy to all foods used in the study, and no self-reported sleep disorders. The diagnosis of T2DM was based on the standards of the American Diabetes Association [106]: fasting plasma glucose ≥126 mg/dL (7.0 mmol/L) or 2-h postprandial plasma glucose ≥200 mg/dL (11.1 mmol/L) during a 75 g oral glucose tolerance test (OGTT) or HbA1c ≥6.5% (48 mmol/mol) or a random plasma glucose ≥200 mg/dL (11.1 mmol/L). We did not include women in the study to avoid confounding factors derived from hormone-elicited changes in carbohydrate metabolism [107, 108].

Exclusion criteria were: use of exogenous insulin, diabetes-related health complications, gastrointestinal disorders or irregular intestinal rhythm (diarrhea or constipation), smoking, and post-meal blood glucose peak of less than 140 mg/dL (7.8 mmol/L). The cut-off value of 140 mg/dL was based on the fact that healthy subjects usually have euglycemic values below this limit [109–111] and because this threshold is commonly used to define a glycemic response as ‘postprandial hyperglycemia’ [106, 112].

Sample size was determined using G*Power software (Dusseldorf University, Germany) [113–115] with a statistical power of 95%, alpha error of 5% (two-tails), and the glucose levels reported by de Carvalho et al. (135.8 ± 20.7 mg/dL) [116], which resulted in a sample size of 19 participants (crossover design). To compensate for possible dropouts, we were added three extra subjects and recruited a total of 22 volunteers.

#### Clinical assessments

During the initial screening visit, participants completed questionnaires about the study’s criteria, diabetes diagnosis, current medications use, health conditions, eating habits and sleeping routine. Body composition and anthropometric data were also assessed. Then, the subjects reported to a clinical laboratory (07:00–8:30 AM) after an overnight fasting (8–12 h) and abstained from their diabetes medication to collect 3 mL of venous blood in a vacutainer tube with no anticoagulant or preservative. Insulin level was measured by electrochemiluminescence (ADVIA, model Centaur, Siemens Healthcare Diagnostics S.A., Brazil) and glucose was determined by the glucose oxidase method (ADVIA, 208 model 2400, Siemens Healthcare Diagnostics S.A., Brazil). Glycated hemoglobin (HbA1c) was quantified by turbidimetric inhibition immunoassay (COBAS, Roche Diagnostics, Brazil) [117]. The homeostatic model assessment-insulin resistance (HOMA-IR) and HOMA-β were calculated to evaluate insulin resistance and pancreatic β cell function, respectively [118].

#### Anthropometry

Height was measured using a stadiometer (Balmak®) fixed to the wall measuring to the nearest 0.1 cm. Weight was assessed using an electronic scale (Ramuza®) with a precision of 50 g. Body fat was determined by bioelectrical impedance (InBody 570, Seoul, South Korea). The circumference of the waist was measured with a precision of 0.1 cm at the midway between the lowest rib and the iliac crest [119, 120].

### Meals

The standardized meal offered during each experimental session was a nutritionally-balanced breakfast showing an energy and macronutrient composition that followed the recommendations of the Dietary Guidelines for Americans for [121]. The breakfast provided 50.0 g available carbohydrate (59%), 10.7 g fat (29%), 10.0 g protein (12%) and 2.4 g of fiber (40 g of toast, 40 g of cheese and 200 mL of peach juice), totaling 337 kcal. The amount of 50 g of carbohydrates is commonly consumed in the breakfast in North America [122] and in other studies that investigated the effect of cinnamon on the glycemic response [123–126].

### Cinnamon treatment

Ground powder of Chinese cinnamon, obtained from the dried inner bark of the tree *Cinnamomum cassia*, also known as aromaticum/aromaticaum [127], was purchased in packages of 50 g sealed by the manufacturer (Kitano, São Bernardo do Campo, Brazil), all from the same batch (Batch: F19BRPP032). The encapsulation was carried out at a compounding pharmacy (Pharmacotécnica, Brasília, Brazil) using a semi-automatic encapsulator to pack 600 mg of powder into ordinary hard gelatin capsules size 00 from the supplier GEMINI (Batch: 190370) [128, 129]. The cinnamon to be taken in the powder form was weighed on a high-precision scale and separated into small plastic cups with a lid. The doses of 3 and 6 g of crude cinnamon contained, respectively, 4.68 and 9.36 kcal (156 kcal/100 g), 0.10 and 0.19 g of protein (3.21%), 0.06 and 0.12 g of lipids (1.98%), 0.94 and 1.88 g of carbohydrates (31.31%), 1.50 and 3.01 g of total fiber (50.11%), 1.46 and 2.91 g of insoluble fiber (48.56%); and 0.05 and 0.09 g of soluble fiber (1.55%) (Table 1).

**Table 1:** Nutrient content of cinnamon cassia powder in 100 g (%), 3 g and 6 g portions.

	100 g	3 g	6 g
Energy (kcal)	156	4.68	9.36
Protein (g)	3.21	0.10	0.19
Lipids (g)	1.98	0.06	0.12
Carbohydrate (g)	31.31	0.94	1.88
Total fiber (g)	50.11	1.50	3.01
Insoluble fiber (g)	48.56	1.46	2.91
Insoluble fiber (% of total fiber)	96.91	.	.
Soluble fiber (g)	1.55	0.05	0.09
Soluble fiber (% of total fiber)	3.09	.	.

Data calculated from Araújo et al. [130].

The dose of 3 g of raw cinnamon was chosen because the intake of the dose of 1 g (capsules of C. cassia) does not influence the post-meal incremental area under the curve (iAUC) in healthy subjects [131], but the dose of 3 g does [132] and because it may be taken twice a day without surpassing the maximum recommended dose of 6 g per day [7, 133]. The dose of 6 g was also tested considering that the metabolic effects of cinnamon have shown to be dose-dependent [15, 134, 135] and because its acute intake has been demonstrated to be safe in studies under laboratory conditions [123, 136].

The choice of *Cinnamomum cassia* was based on the following information: (a) the precise content of nutrients, insoluble and soluble fiber of other varieties is not available, while *C*. *cassia* constitution was determined by accurate food chemical analyses (Table 1) [130]; (b) has the ability to inhibit the carbohydrate-digesting enzyme α-glucosidase [137–140] more intensely than Ceylon cinnamon (*C*. *zeylanicum*) [131, 141]; (c) is a stronger α-amylase inhibitor than other three varieties: Indonesian (*C*. *burmanii*), Vietnamese (*C*. *loureirii*), and Ceylon (*C*. *zeylanicum*) [141] (d) is highly recommended for administration in glycemic and lipid profiles studies [133]; and (e) is the most common commercial cinnamon variety [142] studied for treating TD2M [21, 133, 143–145]. Moreover, the safety of cinnamon cassia long-term intake has been evidenced by its property to improve liver function in animals [146–152] and the lack of reports of toxicity [82–85, 97, 147, 153, 154], even in a dose of 12 g per day taken by subjects for 12 weeks [153] and the presence of compound potentially hepatotoxic such as coumarin [155–159].

We chose the raw form of cinnamon because baking, frying or cooking foods for ≥10 minutes alters nutrient content and functional properties of vegetables: (a) decreasing the concentration of insoluble fiber [160–165]; (b) decreasing the content of several flavonoids and phenolic compounds [57, 166–168]; (c) decreasing the inhibitory activity on the carbohydrate-hydrolyzing enzymes α-amylase and α-glucosidase [55–57, 169, 170]; and (d) increasing starch hydrolysis rate [171–173]. Heating plants also produces the known toxic/carcinogenic polycyclic aromatic hydrocarbons, heterocyclic amines [58–64, 174] and advanced glycation endproducts [65–67].

### Experimental protocol

After refraining from alcohol and exercise for 24 h and fasting for 8–12 h, 19 patients with T2DM underwent five morning experimental sessions (at 7:00–9:00 AM) separated by a washout period of 3–10 d. Each patient randomly consumed a breakfast either alone (Control) or immediately after prior ingestion of 3 or 6 g of raw cinnamon powder mixed with 150 mL water (3gPowder and 6gPowder groups) or prior ingestion of 5 or 10 capsules (600 mg each) with 150 mL water (3gCaps and 6gCaps groups). The maximum time allowed to ingest cinnamon and the standard meal was 15 minutes. Glycemia was measured at fasting (time 0) and postprandially at 15, 30, 45, 60, 90, and 120 min. After approximately 15 min of finishing each breakfast, each participant completed a visual analog scale (VAS) in order to rate palatability scores for visual appeal, smell and pleasantness of taste were assessed, as well as the taste intensity sweetness, saltiness, bitterness, sourness and creaminess [175]. Due to the property of the material, it was impossible for the participants to be blinded. Blinding was performed during the statistical assessment of the outcomes. Patients were requested to withhold their morning medications until the end of the procedures. During the period of participation in the research, the participants were instructed to keep their usual diet and daily physical activities. In addition, they were asked not to consume cinnamon, even for culinary purposes, during their participation in the research.

### Blood glucose

Glycemic response was measured following Wolever’s recommendations and procedures to achieve the highest sensitivity–through capillary finger-stick blood samples [176]. Blood glucose concentration was measured using a glucometer [177–180] (Accu-Chek Active, Roche, Brazil) [181–184] that meets ISO 15197 accuracy criteria (r = 0.998; hexokinase method) [185]. The same approach has been used in studies that evaluated the effect of a single dose of cinnamon on postprandial blood glucose [123, 132, 136, 186], as well as in other similar recent works [183, 187–189].

#### Calculations

Glucose peak rise, also named as “glucose spikes” [73, 190–193], was defined as the maximum amplitude of glucose excursion, i.e., the maximum blood glucose concentration during the 120-minute test minus the fasting concentration [194, 195]. Time-to-peak was the time taken to reach the maximum measured blood glucose [196, 197]. The variation in glucose concentration (Δ blood glucose) was calculated as the post-meal blood glucose level at the different times throughout the glycemic curve minus fasting concentration [195]. Taking into account the association between higher levels of the 2-hour post-meal blood glucose in T2DM patients with complications and with delayed time-to-peak [69, 75, 76, 198], we also compared the 2-hour post-meal blood glucose among groups. The iAUC for glycemic response was calculated using the trapezoidal method, excluding the values below the baseline [199].

### Statistical analysis

Data were tested for normality by the Shapiro-Wilk test and homoscedasticity by Levene’s test. The effect of raw cinnamon on the following parameters was evaluated using one-way repeated measures ANOVA with post hoc Bonferroni test: mean glucose peak rise, Δ_1-hour_ post-meal blood glucose, 1-h post-meal blood glucose, Δ_2-hour_ blood glucose, 2-h post-meal blood glucose and 2-h iAUC. A non-parametric repeated-measures Friedman test was used to compare data on time-to-glucose-peak and palatability. To examine the effects of the different forms and doses of cinnamon at different time points of the post-meal glycemic curve, we performed two-way repeated measures ANOVA using Bonferroni’s post hoc test for multiple comparisons. Data are presented as means with standard error or medians with 25th and 75th percentiles (interquartile range). All analyses were conducted using the Statistical Package for the Social Sciences (SPSS) software (version 21.0). P < 0.05 was considered significant.

## Results

Initially, ninety-three men responded to the announcement and completed the screening visit. Of those, seventy-one were excluded and twenty-two participants met all eligibility criteria and were enrolled in the study. After clinical assessments and anthropometric measurements, three dropouts occurred due to research abandonment (n = 1), change in working hours (n = 1), and change of place residence (n = 1). Finally, nineteen participants completed the full study protocol (Fig 1).

### Participants’ characteristics

The patients’ characteristics are summarized in Table 2. All patients were using oral hypoglycemic medications: nine (47%) took only one medication (eight used metformin and one used gliclazide), five (26%) took metformin plus glibenclamide, two (10%) took metformin plus vildagliptin, two (11%) took metformin-glibenclamide combination plus dapagliflozin, and one subject (5%) took metformin-glibenclamide plus pioglitazone. None of the subjects reported regular use of cinnamon and all of them did not remember the last time they consumed the herb.

**Table 2:** Baseline characteristics of the nineteen participants that completed the study.

Variable	Median (25^th^-75^th^)	Range
**Age (years)**	56 (47.5–57.0)	(38–59)
**Height (cm)**	172.0 (168.3–176.2)	(154.0–192.0)
**Weight (kg)**	89 (81.55–104.99)	(67.60–128.40)
**Body mass index (kg/m²)**	30.44 (27.15–36.37)	(23.10–41.00)
**Body fat percentage**	34.50 (23.60–36.45)	(15.60–46.60)
**Waist circumference (cm)**	106.5 (100.5–117.25)	(85.0–132.0)
**Months of diabetes duration**	49 (30.5–87)	(6–300)
**Fasting venous glucose (mg/dL)**	124.0 (110.5–138.5)	(81–191)
**Fasting insulin (μUI/mL)**	12.80 (11.50–19.00)	(4.00–33.68)
**HbA1c percentage**	7.0 (6.3–7.6)	(5.6–8.3)
**HOMA-IR**	4.20 (3.00–6.67)	(1.20–9.70)
**HOMA-β**	89.40 (55.65–130.05)	(23.70–676.64)

HbA1c: Glycated hemoglobin; HOMA-IR: Homeostasis model assessment of insulin resistance; HOMA-β: Homeostasis model assessment of beta-cell function.

All participants showed blood glucose levels of at least 150 mg/dL (8.3 mmol/L) at 1-hour post-meal and at peak, confirming that the chosen standardized meal and the selected patients were appropriate to induce a glycemic response that exceeds the normal euglycemic level of 140 mg/dL (7.8 mmol/L) and mimics the postprandial hyperglycemia typically experienced by T2DM patients.

### Post-meal blood glucose spikes (peak rise and Δ_1hour_)

**Fig 2:**
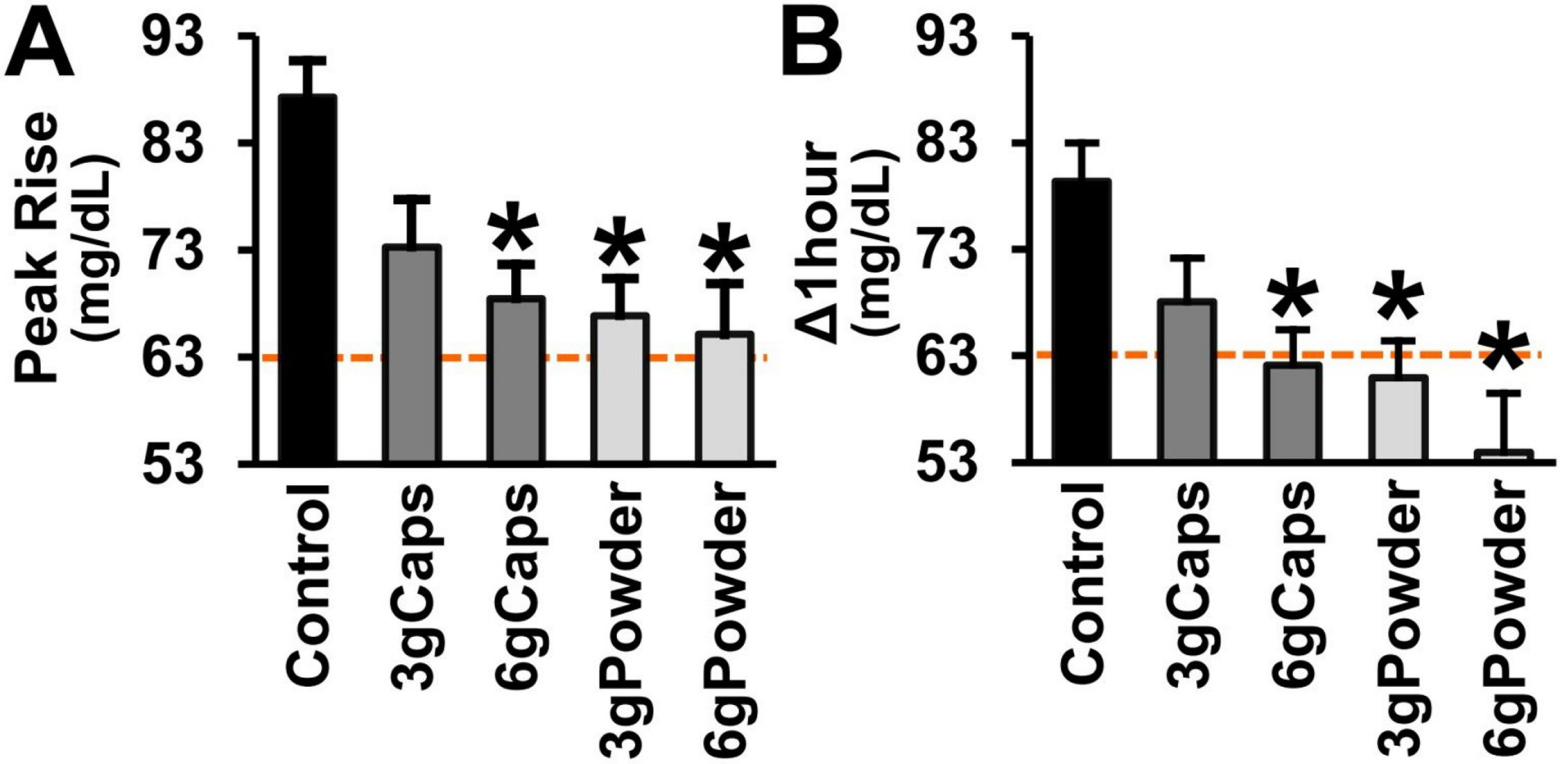
Post-meal blood glucose peak rise and Δ_1hour_ of T2DM patients without or with raw cinnamon. (A) Post-meal mean blood glucose peak rise (mg/dL) and (B) mean difference between 1-hour post-meal blood glucose and fasting blood glucose (Δ1hour) of T2DM men after eating a standardized meal alone (Control) or after prior ingestion of 3 g of raw cinnamon in capsules (3gCaps), 6 g of raw cinnamon in capsules (6gCaps), 3 g of raw cinnamon powder dissolved in water (3gPowder), or 6 g of raw cinnamon powder (6gPowder). The line at the value of +63 mg/dL (+3.5 mmol/L) indicates the maximum mean blood glucose rise achieved by healthy individuals after ingesting 50 g of carbohydrate from 27 tested foods [194]. *p ≤ 0.013 in relation to control.

After the intake of the standardized meal, the fasted patients with T2DM showed a mean peak rise (glucose spike) of +87 mg/dL and a mean post-meal Δ_1-hour_ blood glucose of +79 mg/dL (control group). These values of maximum amplitude of glucose excursion surpass the highest mean blood glucose rise of +63 mg/dL (+3.5 mmol/L) achieved by healthy individuals after ingesting 50 g of carbohydrate from 27 tested foods [194], as indicated in Fig 2 (orange lines).

The acute ingestion of raw cinnamon markedly decreased blood glucose peak rise (one-way RM-ANOVA [F(4,72) = 8.260; p < 0.001]) (Fig 2A). The post hoc multiple comparisons tests (Bonferroni-adjusted) showed that the mean peak rise of the T2DM patients (+87 mg/dL) was significantly reduced by the intake of the dose of 3 g of raw cinnamon in the form powder (-23%; p = 0.003), but not by this dose taken as capsules (p = 0.139) (Fig 2A). The mean peak rise was decreased by the dose of 6 g in both forms, capsules (-22%; p = 0.013) and powder (-25%; p = 0.001) (Fig 2A). There was no difference in mean peak rise between the four cinnamon groups (p > 0.05). The groups 3gCaps, 6gCaps, 3gPowder and 6gPowder showed a mean peak rise of +73 mg/dL, +68 mg/dL, +67 mg/dL and +65 mg/dL, respectively (Fig 2A).

The post-meal level of Δ1-hour blood glucose (+79 mg/dL) was also decreased by the acute intake of raw cinnamon (one-way RM-ANOVA [F(4,72) = 8.947; p < 0.001]) (Fig 2B). The post hoc multiple comparisons tests (Bonferroni-adjusted) showed that the post-meal mean Δ_1-hour_ blood glucose of the fasted T2DM patients after breakfast was decreased by the dose of 3 g of raw cinnamon in the form powder (-22%; p < 0.001), but not by capsules (p = 0.083) (Fig 2B). The Δ_1-hour_ blood glucose was reduced by 6 g of raw cinnamon in both forms, capsules (-23%; p = 0.006) and powder (-32%; p = 0.003). There was no difference in mean Δ_1-hour_ blood glucose between the four cinnamon groups (p > 0.05). The decreases in the mean Δ_1-hour_ blood glucose provided by raw cinnamon were sufficient to achieve values that are proximate to those (+63 mg/dL or +3.5 mmol/L) of healthy individuals (Fig 2). The groups 3gCaps, 6gCaps, 3gPowder and 6gPowder showed a mean Δ_1-hour_ blood glucose of +68 mg/dL, +62 mg/dL, +61 mg/dL and +54 mg/dL, respectively (Fig 2B).

### Post-meal hyperglycemia

**Fig 3:**
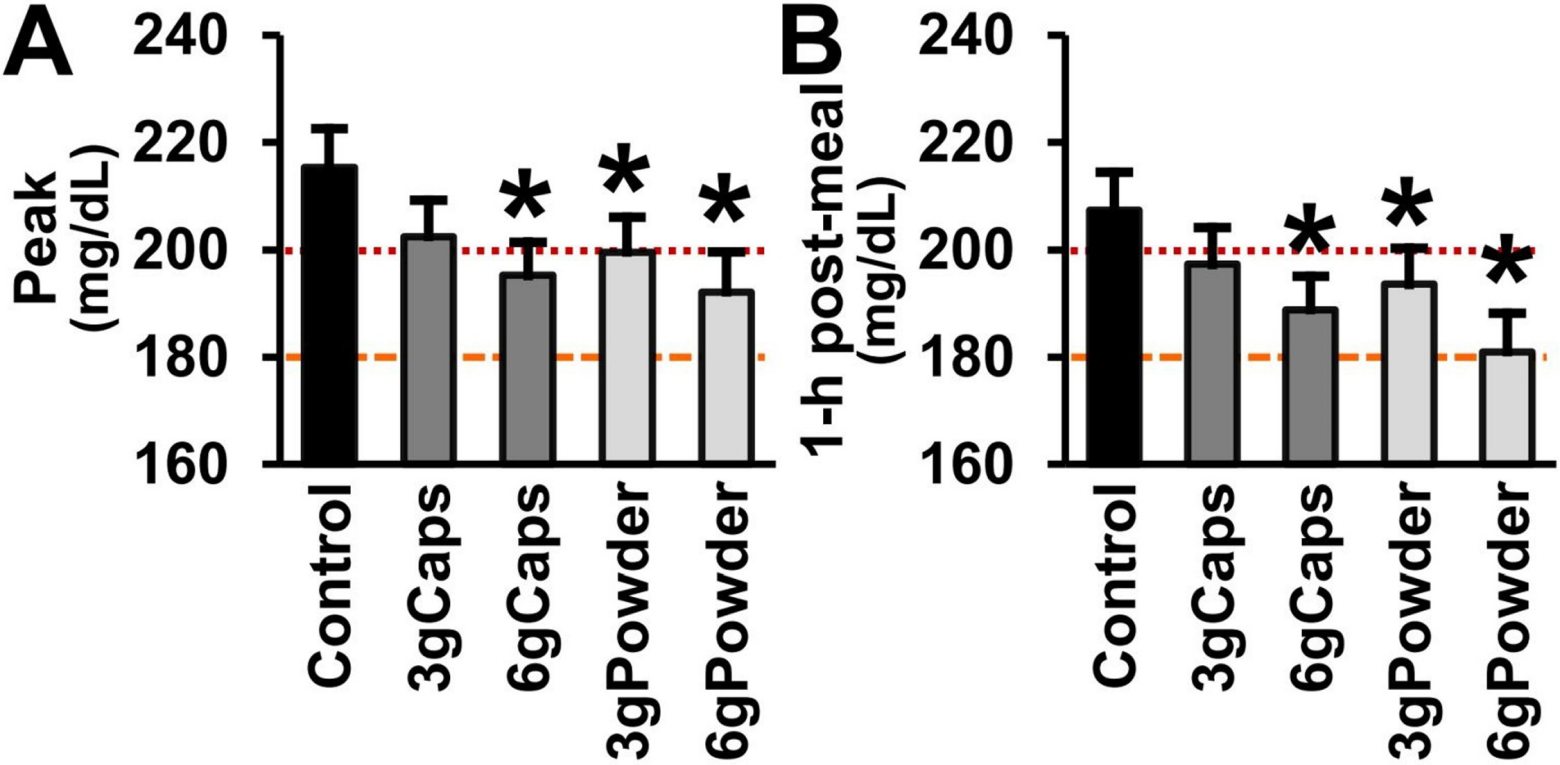
Post-meal blood glucose peak and 1-h post-meal of T2DM patients without or with raw cinnamon. (A) Post-meal mean blood glucose peak (mg/dL) and (B) mean 1-hour post-meal blood glucose (mg/dL) of T2DM men after eating a standardized meal alone (Control) or after prior ingestion of 3 g of raw cinnamon in capsules (3gCaps), 6 g of raw cinnamon in capsules (6gCaps), 3 g of raw cinnamon powder dissolved in water (3gPowder), or 6 g of raw cinnamon powder (6gPowder). The line at the value of 180 mg/dL (10 mmol/L) indicates the level up to which renal glucose reabsorption is preserved at physiological rates [200–202] and insulin therapy is not yet necessary [203, 204]. The line at the value of 200 mg/dL (11.1 mmol/L) indicates the 1-h post-meal threshold used to diagnose T2DM [205] and strongly associated with metabolic disturbances [206]. *p ≤ 0.017 in relation to control.

The mean of the highest blood glucose level and the 1-h post-meal mean blood glucose level of the fasted T2DM patients (control) after eating the breakfast were hyperglycemic (higher than 140 mg/dL; >7.8 mmol/L): 215 mg/dL and 207 mg/dL, respectively. As indicated in Fig 3, these values are higher than the renal threshold of 180 mg/dL (10 mmol/L) up to which glucose reabsorption is preserved at physiological rates [200–202] and insulin therapy is not necessary [203, 204]. They are also higher than the cutoff level of 200 mg/dL (11.1 mmol/L) used to diagnose T2DM [205] and strongly associated with metabolic disturbances [206].

The acute ingestion of raw cinnamon decreased the post-meal hyperglycemic peak (one-way RM-ANOVA [F(4,72) = 5.288; p < 0.001]) (Fig 3A). The post hoc multiple comparisons tests (Bonferroni-adjusted) showed that the mean blood glucose peak of the T2DM patients (215 mg/dL) was significantly reduced by the intake of the dose of 3 g of raw cinnamon in the form powder (-9%; p = 0.009), but not by this dose taken in the form of capsules (p = 0.644) (Fig 3A). The mean blood glucose peak was also decreased by the dose of 6 g in both forms, capsules (-6%; p = 0.016) and powder (-13%; p = 0.003) (Fig 3A). The groups 3gCaps, 6gCaps, 3gPowder and 6gPowder showed a mean peak of 202 mg/dL, 195 mg/dL, 200 mg/dL and 192 mg/dL, respectively (Fig 3A).

The hyperglycemic level of the 1-hour post-meal blood glucose (207 mg/dL) was also decreased by the acute intake of raw cinnamon (one-way RM-ANOVA [F(4,72) = 6.763; p < 0.001]) (Fig 3B). The post hoc multiple comparisons tests (Bonferroni-adjusted) showed that the mean blood glucose level of the T2DM patients at one hour after breakfast was significantly decreased by the dose of 3 g of raw cinnamon in the form powder (-9%; p = 0.017), but not by capsules (p = 0.935) (Fig 3B). The level of 1-h post-meal blood glucose was reduced by 6 g of raw cinnamon in both forms, capsules (-6%; p = 0.013) and powder (-13%; p = 0.001). However, there was no difference in the mean peak and the level of 1-h post-meal blood glucose between the four cinnamon groups (p > 0.05). The groups 3gCaps, 6gCaps, 3gPowder and 6gPowder showed a mean 1-hour post-meal blood glucose of 197 mg/dL, 189 mg/dL, 194 mg/dL and 181 mg/dL, respectively (Fig 3B).

### Time to blood glucose peak

**Fig 4:**
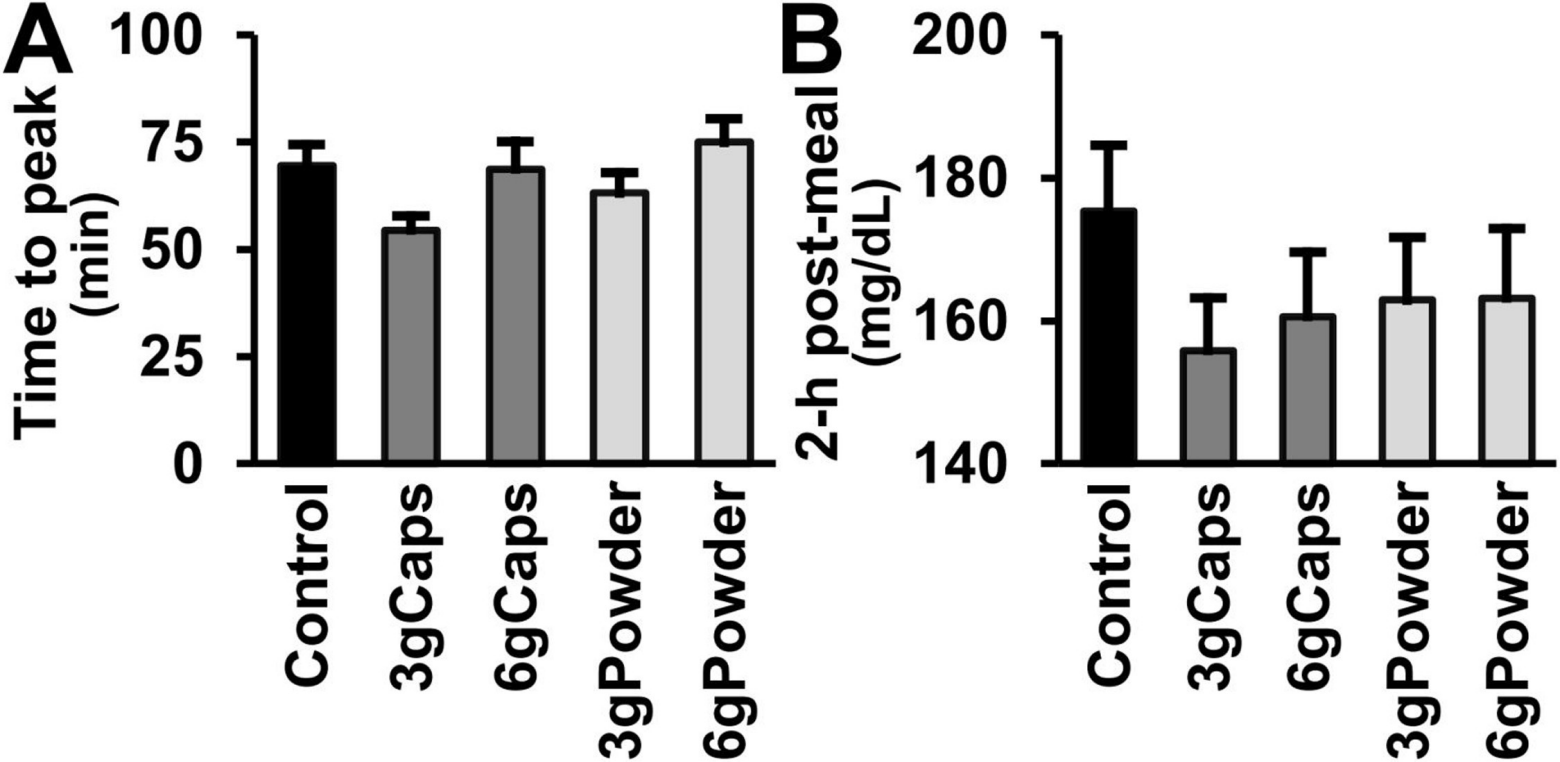
Post-meal time to blood glucose peak and 2-h post-meal of T2DM patients without or with raw cinnamon. (A) Post-meal mean time (min) to reach blood glucose peak and (B) mean 2-hour post-meal blood glucose (mg/dL) of T2DM men after eating a standardized meal alone (Control) or after prior ingestion of 3 g of raw cinnamon in capsules (3gCaps), 6 g of raw cinnamon in capsules (6gCaps), 3 g of raw cinnamon powder dissolved in water (3gPowder), or 6 g of raw cinnamon powder (6gPowder).

The level of blood glucose in the patients with T2DM reached its peak at a mean time of 69.5 minutes after eating breakfast (Control) (Fig 4A). The occurrence of the peak at this time, which is greatly later than the normal time-to-peak of healthy individuals, is an additional marker of insulin resistance in our volunteers [69–71]. In all tests, the highest mean glycemic levels were observed at the 1-hour post-meal time. None of the doses or forms of cinnamon intake changed the time-to-peak in relation to the standard meal alone (Friedman test; p ≥ 0.159).

### 2-h post-meal blood glucose

In line with the delay in the time-to-peak of T2DM patients, the mean blood glucose level at two hours (175 mg/dL) after the intake of the standardized meal containing 50 g of complex carbohydrate was still higher (+47 mg/dL) than fasting (Fig 4B). The mean 2-h post-meal of 175 mg/dL surpassed the threshold of 166 mg/dL (9.2 mmol/L) used after a post-load of 75 g of glucose to diagnose impaired glucose tolerance (IGT) and impaired fasting blood glucose (IFG) [207]. In contrast, all cinnamon groups showed mean levels of 2-h post-meal blood glucose lower than this mark: the groups 3gCaps, 6gCaps, 3gPowder, and 6gPowder showed a mean 2-h post-meal blood glucose of 156 mg/dL, 161 mg/dL, 163 mg/dL, and 163 mg/dL, respectively (Fig 4B). However, there was no significant difference in the mean 2-h post-meal blood glucose among groups (RM-ANOVA; [F(4,72) = 2.476; p = 0.052]).

### Effects of dose and capsule

The two-way ANOVA with repeated measures showed that the form (capsule or powder dissolved in water) that T2DM patients used to intake raw cinnamon exerted a significant effect (interaction between the factors ‘form’ and ‘time’) on their blood glucose levels throughout 120 minutes [F(2.187, 196.796) = 3.972; p = 0.017]. Post-hoc analysis showed that the mean blood glucose level was significantly decreased by raw cinnamon ingested in the form of powder at 30 min (p = 0.013) at 45 min (p = 0.042). The effect of capsule did not change blood glucose levels (p ≥ 0.159) although it did not differ from the effect of powder (p ≥ 0.168). The dose (3 g or 6 g) of raw cinnamon did not exerte a significant effect on blood glucose levels at the different times, since the interaction between ‘dose’ and ‘time’ did not reach statistical significance (p = 0.127) as well as between ‘form’, ‘dose’ and ‘time’ (p = 0.993).

The post-meal values of Δ blood glucose after the fasted T2DM patients ingested breakfast were significantly changed along the glycemic curve (interaction between the factors ‘form’ and ‘time’) by the form used to intake raw cinnamon [F(2.717, 244.498) = 3.307; p = 0.025]. The levels of Δ blood glucose were significantly decreased by the intake of raw cinnamon in the form of powder at 15 min (p = 0.001), at 30 min (p < 0.001), at 45 min (p < 0.001), at 60 min (p < 0.001), and at 90 min (p = 0.013) and in the form of capsules at 15 min (p = 0.020), 60 min (p = 0.016), and 90 min (p = 0.004). The decreases in Δ blood glucose levels caused by the form of powder were significantly greater than by the form of capsule at 30 min (p = 0.002) and at 45 min (p = 0.003). The effect of the interaction between ‘dose’ and ‘time’ (p = 0.204) and of the interaction between ‘dose’, ‘form’, and ‘time’ on Δ blood glucose levels was not significant (p = 0.978).

### Glycemic response curve and iAUC

**Fig 5:**
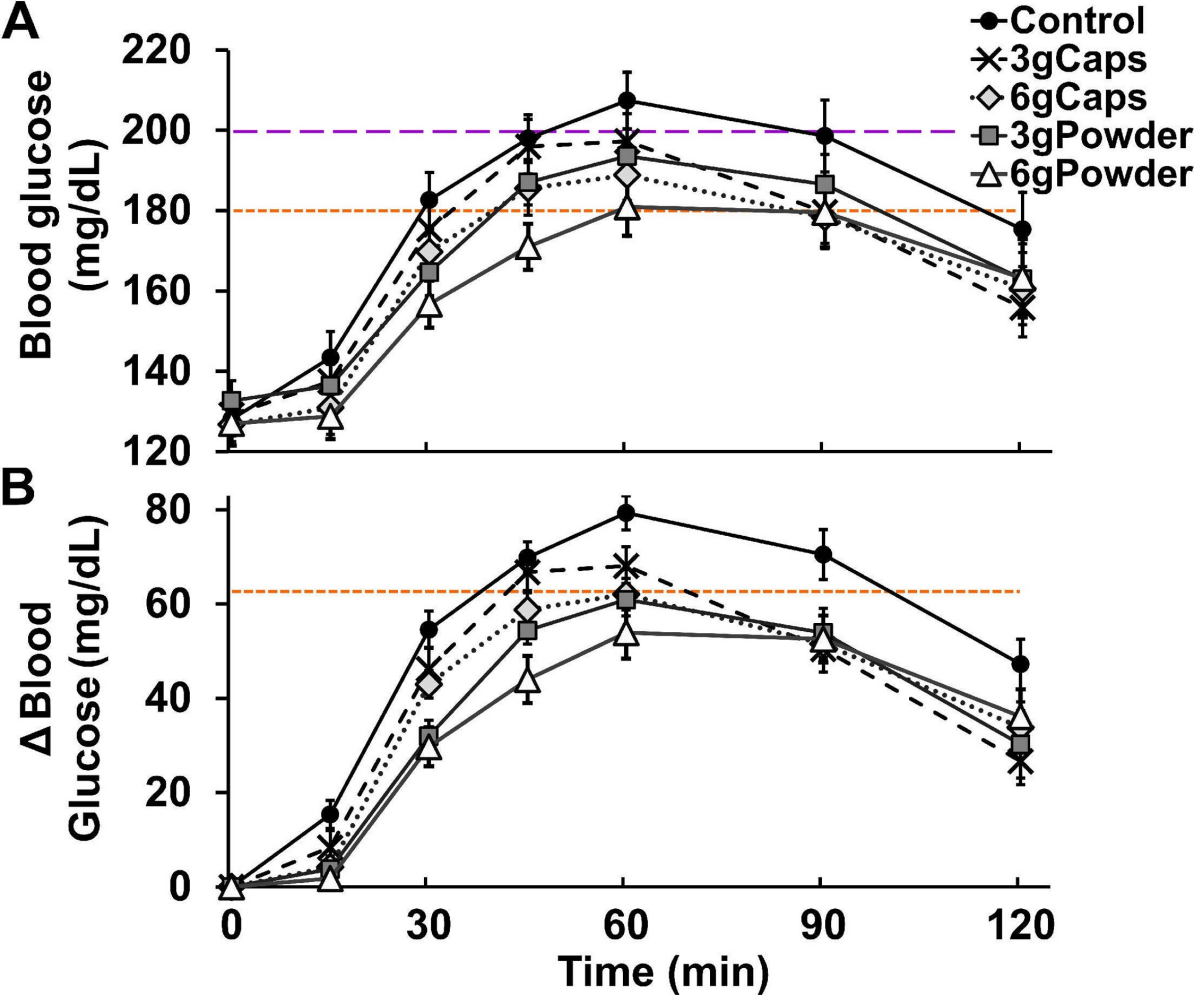
Post-meal glycemic curve of T2DM patients without or with raw cinnamon. (A) Mean blood glucose (mg/dL) and (B) mean Δ blood glucose (difference between post-meal blood glucose and fasting blood glucose) of T2DM men throughout 120 minutes after eating a standardized meal alone (Control) or after prior ingestion of 3 g of raw cinnamon in capsules (3gCaps), 6 g of raw cinnamon in capsules (6gCaps), 3 g of raw cinnamon powder dissolved in water (3gPowder), or 6 g of raw cinnamon powder (6gPowder). The line at the value of 180 mg/dL (10 mmol/L) indicates the level up to which renal glucose reabsorption is preserved at physiological rates [200–202] and insulin therapy is not yet necessary [203, 204]. The line at the value of 200 mg/dL (11.1 mmol/L) indicates the 1-h post-meal threshold used to diagnose T2DM [205] and strongly associated with metabolic disturbances [206]. The line at the value of 63 mg/dL (3.5 mmol/L) indicates the maximum mean blood glucose rise achieved by healthy individuals after ingesting 50 g of carbohydrate from 27 tested foods [194]. The post-meal blood glucose levels along the glycemic curve were significantly decreased by raw cinnamon ingested in the form of powder (p ≤ 0.042), independently of the dose (p > 0.05), but not in the form of capsule (p ≥ 0.159) (two-way ANOVA with repeated measures). The levels of post-meal Δ blood glucose along the curve were significantly decreased by raw cinnamon ingested in the form of powder (p ≤ 0.013) and in the form of capsule (p ≤ 0.020), independently of the dose (p > 0.05), and the decreases caused by the form of powder were significantly stronger than by the form of capsule (p ≤ 0.003).

The glycemic response curve of the participants in the present study showed a shape after eating breakfast (Control) that is typical of patients with T2DM. The Fig 5 shows that, in contrast to the glycemic curve of healthy individuals, the time to blood glucose peak occurred more than 100% later in our T2DM participants [69–71]. Their blood glucose level after peak also showed a slight decay (opposing to the normal rapid drop) and the level of the 2-h post-meal blood glucose was still elevated at a hyperglycemic level (>140 mg//dL) [69–71]. The intake of powder of raw cinnamon dissolved in water flattened the glycemic curve without changing the shape, as evidenced by the above reported results of time-to-peak and 2-h post-meal blood glucose.

**Fig 6:**
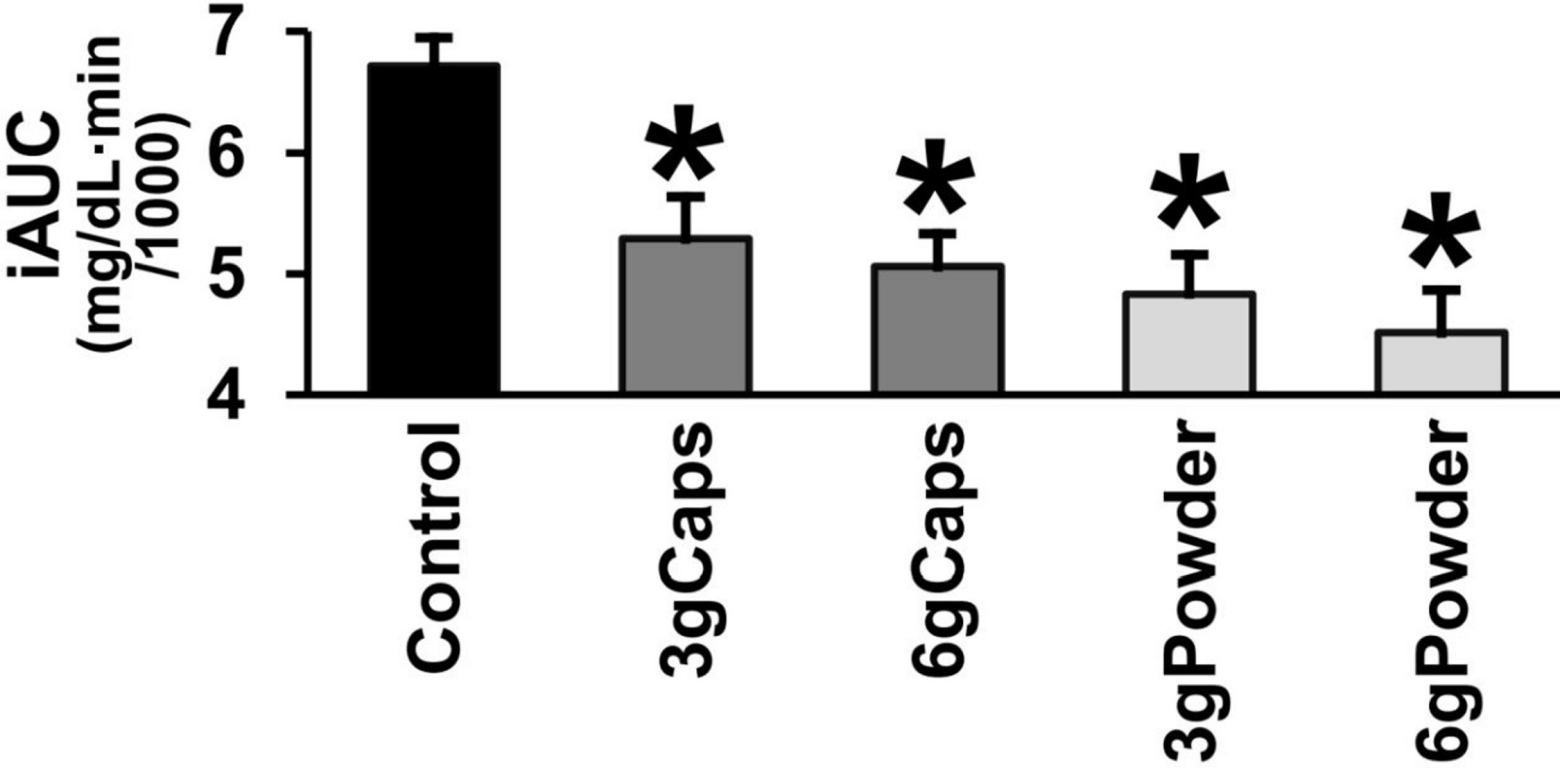
Post-meal incremental area under the glycemic response curve (iAUC) of T2DM patients without or with raw cinnamon. Post-meal incremental area under the glycemic response curve (iAUC) of T2DM men after eating a standardized meal alone (Control) or after prior ingestion of 3 g of raw cinnamon in capsules (3gCaps), 6 g of raw cinnamon in capsules (6gCaps), 3 g of raw cinnamon powder dissolved in water (3gPowder), or 6 g of raw cinnamon powder (6gPowder). *p ≤ 0.012 in relation to control.

As a consequence of this general flattening, raw cinnamon caused a marked decrease in iAUC (Fig 6). The iAUC of the patients with T2DM after the intake of the breakfast was significantly decreased by the acute ingestion of raw cinnamon (one-way RM-ANOVA [F(4,72) = 14.216; p < 0.001]). The mean iAUC was lower in all raw cinnamon groups in comparison to control: 3gCaps (-21%; p = 0.012), 6gCaps (-25%; p < 0.001), 3gPowder (-28%; p < 0.001), and 6gPowder (-33%; p < 0.001) (Fig 6).

### Markers of palatability and taste intensity

The prior ingestion of 3 or 6 g of raw cinnamon in the form of capsules or powder dissolved in water did not change any of the rates of palatability or taste intensity of the standardized meal. No significant differences between groups (p ≥ 0.80) were observed: visual appeal, smell, pleasantness of taste, sweetness, saltiness, bitterness, sourness and creaminess (Table 3).

**Table 3:** Taste perception ratings (median and 25^th^ -75^th^ interquartile) of T2DM men after eating breakfast without (control) or after prior ingestion of raw cinnamon in the forms/doses of 3 g capsules, 3 g powder, 6 g capsules or 6 g powder.

		Control	3gCaps	6gCaps	3gPowder	6gPowder
**PALATABILITY**	**Visual appeal**	8.00	7.80	7.40	7.80	7.30
		(6.65–9.45)	(7.05–8.90)	(6.60–9.45)	(6.75–9.25)	(6.85–9.35)
	**Smell**	7.80	7.30	7.90	7.30	6.90
		(6.65–9.10)	(6.50–8.85)	(6.20–9.45)	(6.65–8.90)	(5.60–8.70)
	**Pleasantness of taste**	7.50	8.00	7.00	7.50	7.50
		(6.85–8.85)	(6.75–9.20)	(6.40–8.55)	(6.40–9.00)	(5.80–9.25)
**TASTE INTENSITY**	**Sweetness**	5.30	5.20	5.00	5.30	5.40
		(3.85–6.95)	(2.95–6.10)	(3.15–6.70)	(2.70–6.90)	(2.45–7.15)
	**Saltiness**	2.10	4.00	3.30	2.30	4.20
		(1.35–5.20)	(1.75–5.25)	(1.60–4.85)	(1.55–5.20)	(1.75–5.25)
	**Bitterness**	1.80	1.90	2.10	2.30	2.20
		(0.50–3.20)	(0.65–4.55)	(0.95–3.55)	(0.35–7.80)	(1.50–6.70)
	**Sourness**	1.80	2.20	2.70	2.40	2.40
		(0.75–3.15)	(1.20–4.30)	(1.55–3.90)	(0.90–4.50)	(1.75–4.70)
	**Creaminess**	7.80	7.30	7.70	7.40	7.10
		(6.25–9.30)	(6.35–8.95)	(5.90–9.10)	(6.55–8.85)	(6.40–8.50)

## Discussion

The present study investigated the effect of a single dose of raw cinnamon on the blood glucose increase of patients with T2DM after the intake of a nutritionally balanced meal containing complex carbohydrates. After a breakfast providing 50 g carbohydrates, all participants showed post-meal blood glucose levels that exceeded normal euglycemic values and achieved the postprandial hyperglycemia commonly experienced by T2DM patients. The ingestion of raw cinnamon powder dissolved in water, independently of the dose, decreased the meal-induced large glucose spike (peak-rise and Δ_1-hour_ blood glucose) and the hyperglycemic blood glucose peak. When the herb was taken in the form of capsules, these anti-hyperglycemic effects were lost or significantly diminished. The intake of raw cinnamon did not change time-to-peak or the 2-h post-meal blood glucose, but flattened the glycemic curve without changing the shape that is typical of T2DM patients, as evidenced by the decrease in iAUC. Lastly, another new finding was that the palatability of a standard meal is not affected by previous intake of cinnamon dissolved in water.

### Acute effect of raw cinnamon on post-meal glucose spikes

Before this study, research has exclusively used non-diabetic populations to investigate the acute effect of cinnamon on the post-meal blood glucose elevation in a real-life situation (i.e., in response to the ingestion of complex carbohydrates) [123, 131, 136, 208, 209]. These studies reported glucose excursions that are within the normal range, with the highest mean Δ of approximately +45–62 mg/dL [210], which do not reach harmful levels [211]. In contrast, abrupt hyperglycemic ‘spikes’ observed in T2DM patients are highly deleterious and even more damaging than steady hyperglycemia [212–216]. In the present study, a balanced meal providing 50 g of carbohydrates caused mean values of peak rise of 87 mg/dL and Δ_60-0min_ blood glucose of +79 mg/dL in patients with T2DM, values that are close to those from T2DM patients eating controlled meals, e.g., +79 mg/dL and +75 mg/dL [217]. These numbers exceed, by far, normal values of healthy individuals after eating breakfasts [210] and food such as white bread [23, 199, 218–221], white rice [222], brown rice [223], banana [224], and glucose solution [188, 225]. Hence, the present study is the first to assess the potential of raw cinnamon to acutely reduce the large glycemic response that T2DM patients show after a regular meal. A single dose of raw cinnamon taken by T2DM patients caused decreases of 22–32% in the mean post-meal peak rise and the 1-hour post-meal Δ_60-0min_ blood glucose. One mechanism that underlie the deleterious effects of glucose spikes is the formation of free radicals and other reactive oxygen species (ROS) associated with the drastic shifts in glucose availability [226–232]. Indeed, there are a number of similarities between abrupt hyperglycemic excursions and episodes of ischemia/reperfusion, which are associated with extensive oxidative damage [229, 233–238]. Blood glucose spikes also promote an overproduction of ROS [239], oxidative stress [72, 240–244], and glucotoxicity [245–247]. Therefore, the post-meal hyperglycemic glucose spikes from our patients were down to the non-harmful physiological spikes seen in healthy humans [211] by simply drinking a glass of water with raw cinnamon before breakfast.

According to the American Diabetes Association, a higher ‘glycemic burden’ is suggested to explain higher levels of metabolic products of glucose-induced non-enzymatic reactions in T2DM patients [106] that may be monitored by measuring glycated albumin [248–251], fructosamine [248, 252–254], glycated β-lipoprotein [248, 255], glycated LDL [256–258], glycated HDL [259], and/or glycosylated hemoglobin [119, 260]. In accordance to this, the suppression in post-meal spikes for some weeks indirectly increases insulin sensitivity [193, 261–267]. Therefore, it is plausible to expect that the suppression of the post-meal glucose peak rises in patients with T2DM by raw cinnamon may decrease the levels of these markers. In the same way, considering that interventions which decrease glucose spikes diminish oxidative stress [242, 243] by inhibiting the overproduction of ROS [240, 241, 268], the addition of raw cinnamon in the diet of patients with T2DM, taken before the main meals, may also reduce the levels of the ROS-modified particule/biomolecule oxidized LDL [269–271] and carbamylated albumin [272]. Still, these speculations warrant further research.

### Acute effect of raw cinnamon on post-meal hyperglycemia

At a first glance, the decreases in the blood glucose peak caused by the intake of raw cinnamon in the form of powder (-32%) observed in the present study seem similar to those observed in other studies with participants without diabetes; however, post-meal euglycemic peaks of healthy subjects, within the physiological range, i.e., <140 mg/dL (7.8 mmol/L) [111, 210], do not have deleterious effects [211, 273]. On the other hand, postprandial hyperglycemic levels as high as 155 mg/dL (8.6 mmol/L) at 1-h are associated with T2DM-related complications, which worsen as the degree of hyperglycemia increases [200, 274–276]. In the present study, each participant showed blood glucose levels of at least 150 mg/dL (8.3 mmol/L) at 1-hour post-meal and at peak, confirming that all of them experienced postprandial hyperglycemia [112]. The values of 207 mg/dL at 1-hour post-meal and 211 mg/dL at peak blood glucose level measured in the present study are classified as ‘very high’ hyperglycemia (>160 mg/dL) [109] and are very similar to those reported previously for T2DM patients [277]. Consequently, the decreases that were observed in the hyperglycemic post-meal blood glucose peaks in patients with T2DM caused by raw cinnamon powder is another new finding of the present study.

### Meal-induced time-to-glucose peak and 2-h post-meal blood glucose

The intake of raw cinnamon before breakfast did not change the post-meal time-to-glucose-peak. This lack of effect greatly differs from the delays of up to 65 min in the blood glucose rise caused by fat [278] and viscous soluble fibers, such as guar gum [279], psyllium [280], and oat beta-glucans [197, 281, 282]. Considering that this delay is due to their property to slow gastric emptying and/or to prolong gastrointestinal transit time, the absence of effect of cinnamon on time-to-peak indicates that this spice would not retard gastric emptying or slow intestinal transit time. This is supported by the observations that insoluble fibers do not slow gastric emptying or prolong gastrointestinal transit time [283–285] and more than half of the weight of raw cinnamon is actually dietary fiber, which is made up of almost exclusively (97% of total fibre content) water insoluble fiber [130]. In contrast to the postulated gastric emptying effect of cinnamon [23, 145, 286], the decrease in post-meal blood glucose curve caused by raw cinnamon is very similar to those caused by amylase/glycosidase inhibitors, which do not prolong the gastrointestinal transit time [277, 283–285, 287].

The idea that cinnamon would delay digestion comes from studies with animals that received cinnamaldehyde; however, the dose used (250 mg/kg) [288] is impossible to achieve by the ingestion of natural cinnamon [289]. The concentration of cinnamaldehyde in powdered cinnamon cassia is estimated between 1.8 mg/g [290] and 57 mg/g of powder [289]. Actually, in rats, cinnamon has an opposite effect. It promotes peristaltic propulsion and accelerates both gastric emptying and reduces gastrointestinal transit time, showing a laxative effect against constipation [82]. The lack of change in the post-meal time-to-peak by raw cinnamon ingestion corroborates the findings that gastrointestinal transit time in humans is not altered by cinnamon [124] or is only slightly reduced (-7%) [123]. Since longer time to reach blood glucose peak is associated with more diabetes-related markers [70, 291], the absence of effect of cinnamon on this parameter may be beneficial.

Two hours after the intake of a balanced meal, our patients with T2DM showed higher blood glucose means than fasting levels. This response is very similar to that of diabetic patients of the majority of the studies investigating post-meal glycemic curves [277, 292]. Among our five experimental groups, those that did not ingest cinnamon (control) showed the highest 2-h post-meal mean of absolute of 175 mg/dL, exceeding the recommended postprandial threshold of 160 mg/dL (8.89 mmol/L) for optimal glycemic control [194, 276, 293, 294]. Larger values of 2-hour post-meal blood glucose are associated with more severe diabetic complications [69, 75, 76] and delays in the time-to-glucose-peak [71]. In healthy individuals, the level of postprandial blood glucose normally returns to fasting values, even after a load of 75 g of glucose in solution [225]. Here, the prior intake of raw cinnamon resulted in 2-hour post-meal blood glucose means that were lower or near the aforementioned threshold of 160 mg/dL. Therefore, raw cinnamon improved also this diabetes-associated parameter.

### Effects of hard gelatin capsules

The decrease in the meal-induced large blood glucose peak-rise caused by the ingestion of raw cinnamon powder dissolved in water was lost when this herb was taken in the form of capsules. This result contradicts the assumption that common hard gelatin capsules would be promptly dissolved after being swallowed [295–297] and indicates that the high use of this formulation to administrate medicinal plants [41–45] might reduce the desired benefits of their consumption. However, our results confirm some findings of the few articles in the literature that directly compared the absorption/pharmacokinetics of some drugs between these two formulations: capsules versus solution [298, 299]. For example, the maximal blood concentration of an anti-allergic medication taken diluted in water is decreased by 33% when taken through capsules [300]. Among studies that investigated molecules of natural origin, one showed that the absorption of caffeine in the form of chewing gum is decreased when it is taken through capsules [50] and that the bioavailability of polyphenols and other bioactive molecules through the consumption of tea may also be lowered when the herb is ingested as capsules [51–53, 155, 301, 302]. Moreover, the metabolic change caused by red pepper ingested orally is stronger than consumed in capsule form [303–305]. These data may be explained by the fact that rupture and disintegration of the shell of standard gelatin capsules are processes required to release their bioactive compounds [43, 128, 129, 306–309]. In accordance to this, it was shown that the disintegration of capsules containing herbal products (Ginkgo leaf) may fail [310]. To our knowledge, the present study is the first that realized a direct comparison between the formulations of capsule versus solution to deliver a plant powder and to obtain its anti-hyperglycemic effect. The results showed that the anti-hyperglycemic effect of raw cinnamon powder is significantly weakened when the plant is ingested through ordinary hard gelatin capsules formulation.

### Acute effect of raw cinnamon on iAUC

In previous studies that showed that the intake of cinnamon or its extract [209] decreases the post-meal glycemic response, the participants ingested meals showing medium [123] and high [131, 136] glycemic index [194, 311], which produced iAUCs that are normally observed in a real-world post-breakfast circumstance [173, 312]. In contrast, only a slight (but still significant) decrease of 7% in the iAUC was reported after consuming a meal with a very low glycemic index [132].

Herein, the intake of the dose of 3 g of raw cinnamon decreased the mean post-meal 1-hour blood glucose only in the form of powder. The dose of 6 g diminished the control level of 207 mg/dL to significantly lower levels, reaching 181 mg/dL. Another noteworthy effect of raw cinnamon ingestion was the reduction in iAUC_>180mg/dL_. Recently developed approaches measure specifically the iAUC above the threshold 180 mg/dL (iAUC_>180mg/dL_) (associated with metabolic disturbances and mortality) [251, 313] and the time spent in the range of 181–250 mg/dL (TIR_181-250_) through continuous glucose monitoring (CGM) [204]. Here, the mean iAUC_>180mg/dL_ of our T2DM patients (as may be seen in the glycemic curve) approached 0 in patients that had ingested 6 g of raw cinnamon mixed with water. Similarly, the use of CGM by our T2DM patients during the post-meal hours [314] revealed the disappearance of the TIR_181-250_ by the prior intake of raw cinnamon. This acute antihyperglycemic effect exerted by raw cinnamon indicates its usefulness for T2DM patients to follow the recommendation of avoiding post-meal blood glucose levels of 180 mg/dL (10 mmol/L) [203, 204, 315]. Theoretically, the availability of this antihyperglycemic herb to diabetic patients could contribute to rapid improvements in glycemic control since partial and complete remission are achieved when they are asked to monitor their plasma glucose and to avoid ‘anormal’ levels higher than 140 mg/dL (7.8 mmol/L) [316].

These data were produced during experimental human studies that followed the methods recommended to investigate glycemic response, such as the use of the standard portion size of 50 g carbohydrate in a controlled laboratory environment [317], allowing rigorous control of variables and confounding factors [318, 319]. These tightly controlled conditions confers more precision, accuracy, and degree of sensitivity in relation to free-living interventional studies [320–323] that investigate the chronic effects of long-term dietary modifications [324–328]. Taken together with previous research, our results demonstrated that, after common meals, the acute intake of raw cinnamon suppresses not only the postprandial normal iAUC in healthy subjects, but also the hyperglycemic iAUC in T2DM patients.

### Mechanistic considerations

As discussed above, slowing gastric emptying is unlikely the mechanism associated with the observed antidiabetic property of raw cinnamon in this study. One common postulated mechanism of action of cinnamon is an increase in peripheral glucose uptake through increased insulin sensitivity [2, 15, 37, 38, 84, 104, 291, 329–349]. This explanation is based on data from in vitro studies using isolated cells, which showed that cinnamon induced changes in the expression of insulin-sensitive glucose transporter type 4 (GLUT-4), peroxisome proliferator-activated receptors (PPARs), glycogen-associated protein kinase B (Akt) signaling pathway and/or adenosine monophosphate-activated protein kinase (AMPK) [26, 35, 91, 142, 340, 347, 350–358]. However, a single oral dose of cinnamon given to rats [359, 360] and humans does not decrease steady blood glucose concentrations [124, 236], and there are no reports of hypoglycemia in humans with the use of any type of cinnamon [361], contrasting insulin sensitizers. Differently from cinnamon, a single dose of the following molecules and plants (known to act by increasing insulin sensitivity) cause a rapid drop in blood glucose level: pioglitazone [362–365]; metformin [142, 366–369]; glibenclamide [142, 370]; and extracts of Black tea (Camellia sinensis) and of Peniocereus greggii [142, 366]. The molecular changes involved in these hypoglycemic effects occur very rapidly [362, 363, 371, 372], for example, a large activation of AMPK in tissues of normal rats is observed 30 min after a single dose of thiazolidinedione [373]. Cinnamon also does not cause any acute change in the glycemic response induced by a glucose load during an OGTT in rats [374] and in mice [142]. In humans, several studies showed that the high iAUC elicited by the ingestion of glucose solution is not affected by ingestion of different cinnamon varieties: *C*. *cassia* [375–377], *C*. *burmannii* [186, 378], and *C*. *verum* [379]. In one of these studies, the intake of cinnamon by patients with T2DM, whose mean blood glucose peak reached 327 mg/dL (18.19 mmol/L), had no effect on their post-load hyperglycemia [378]. Only one research group observed a small attenuation (-7%) in the post-glucose load iAUC in humans caused by cinnamon intake [380, 381]. The absence of effect of cinnamon on the OGTT’s glycemic curves also refutes another proposed mechanism: the inhibition of intestinal glucose absorption transporters (SGLT1 and GLUT2) [2, 38, 290, 360, 382–384]. In contrast to cinnamon, a single dose of SGLT1-inhibiting drugs (e.g., canagliflozin) and extracts from plants such as Guava (Psidium Guajava) and Salvia polystachya clearly cause an acute suppression in the OGTT’s iAUC [385, 386]. Only in vitro experiments demonstrated that high concentrations of cinnamon could diminish the absorption of glucose [387–389]. These data support that a direct influence of cinnamon on tissue’s insulin sensitivity or on intestinal absorption is not responsible for its antidiabetic property.

To support the claim that the cinnamon’s antidiabetic effect comes from its ability to enhance insulin sensitivity, some authors cite the molecular modifications (e.g., GLUT-4, PPARs, Akt and AMPK) that are observed with the long-term consumption of this plant [47, 147, 350, 352, 360, 390–397]; however, these changes are the same indirect outcomes of other interventions lasting two or more weeks that reduce glucotoxicity through decreasing glucose excursions [126, 193, 264–266, 398–404] and calorie restriction [267, 405–408]. Besides increasing insulin sensitivity, the simple inhibition for some weeks of carbohydrate digestion in the gastrointestinal tract, without direct systemic actions [409–413], cause increase in AMPK level in tissues [267, 414] and GLUT4 protein and glucose transport [415]. These responses are in accordance with pleiotropic effects of suppressing post-meal glucose spikes [192, 267, 401, 413, 414, 416–426]. Moreover, the markers of insulin sensitivity after the beginning of cinnamon consumption increase only gradually [360, 427], showing the same time-dependent effect of inhibitors of carbohydrate digestion [261, 267, 423, 428–432]. Another distinguishing feature of cinnamon is that the administration of its extract for up to 15 weeks does not cause any effect in glucose tolerance and in GLUT4 level in normal mice and rats, differing from diabetic animals [350, 352, 392]. These long-term effects from cinnamon use contrast the rapid effects of insulin sensitizers and support the explanation that this herbal medicine promotes insulin sensitivity indirectly in a similar manner to compounds whose main property is to attenuate postprandial blood glucose response [265, 266, 398–401].

The absence of effect of cinnamon on glucose solution-induced hyperglycemia greatly differs from the robust decreases (21%-46%) in the glycemic responses observed in humans who had consumed complex carbohydrates in a balanced meal. A difference between these two conditions is that the glycemic response artificially elicited by a glucose solution skips the crucial step of digestion of complex carbohydrates of a normal diet [433–435], allowing the adequate evaluation of glucose metabolism without the strong influence of digestion in the postprandial hyperglycemia [399, 400, 436]. These contrasting results are the same observed with the intake of acarbose [142, 374], which inhibits carbohydrate-hydrolyzing enzymes (alpha-amylases and alpha-glucosidases) with minimal systemic absorption [409–413]; this antihyperglycemic agent does not cause any change in the glycemic response promoted by glucose solution, but greatly decreases the increase in glycemia induced by complex carbohydrates [366, 367, 437, 438]. This similarity indicates that the main mechanism by which cinnamon would exert an antidiabetic effect is by its acarbose-like property to inhibit the activity of carbohydrate-hydrolyzing enzymes.

#### Inhibitory activity on the carbohydrate-hydrolyzing enzymes

Several pieces of evidence point towards the inhibition of carbohydrate-hydrolyzing enzymes as the mechanism underlying the antihyperglycemic effect of cinnamon. The notorious suppression of meal-induced hyperglycemia (present study and other studies) is the same observed in oral sucrose/starch tolerance tests (OSTT). While the ingestion of cinnamon caused a strong decrease in the iAUC during OSTT experiments with rats [374] and mice [142], no change occurred in the iAUC during OGTT [142, 374]. In fact, all cinnamon varieties possess this property of inhibiting carbohydrate digestion [439]. Animal and in vitro studies have shown that cinnamon inhibits the activity of pancreatic amylase [140, 141, 170, 440–443], salivary amylase [444, 445] and intestinal glucosidases [138, 139, 141, 356, 374], more specifically of the disaccharidases sucrase and maltase [137, 374, 440, 446, 447]. *Cinnamomum cassia* and *C*. *burmanii* are more potent inhibitors of glucosidase activity than acarbose and *C*. *zeylanicum* [141], whose mode of inhibition is similar to acarbose [209, 374]. In line with this, cinnamon cassia generally exerts a more pronounced inhibition of carbohydrate digestion in vitro than other medicinal plants [198, 448–451]. Considering that cinnamon’s inhibitory properties are dose-dependent [137, 138, 351, 374, 441, 452], our finding that the glucose peak rise was inhibited by capsules containing raw cinnamon only the large amount of 6 g classified as a high dose (≥6 g/day) [84], but not by the medium dose of 3 g [15], corroborates these data and indicates that a high dose of encapsulated raw cinnamon cassia may be necessary to promote the effect of a medium dose mixed with water. Another finding from the present study that may help to understand the cinnamon’s antidiabetic mechanisms is that the degree of the raw cinnamon’s suppression of the post-meal hyperglycemia observed in our patients was similar to that seen in healthy individuals [131, 136] despite the fact that T2DM patients show lower amylase activity than healthy subjects [453–455]. This indicates that the antihyperglycemic effect of raw cinnamon in T2DM patients could be exerted mainly through the inhibition of alpha-glucosidase activity.

The inhibitory property of raw cinnamon is in agreement with the expected effect of its main constituent, insoluble fiber [130], which inhibits alpha-amylase and alpha-glycosidase in both forms, purified [456–458] or naturally in food [459–463]. This may explain the inverse relationship between the intake of insoluble fiber in the diet and the risk of diabetes [464] and metabolic syndrome [465]. The dose of insoluble fiber given in the present study through raw cinnamon (2.9 g) was sufficient to inhibit glycemic responses in T2DM patients, since it corresponds to more than half of the doses known to exert a post-meal antihyperglycemic effect in patients with T2DM [68, 466]. Studies on the effect of insoluble fiber often use wheat bran, which has insoluble fiber as its main constituent and a nutrient content that is very similar to cinnamon [460]. In agreement with this, the addition of insoluble fiber through wheat bran (a cinnamon-similar food) to the diet promoted glycemic control [461, 467], as well as in the form of whole grain and cereal fiber [459, 462, 468–470]. The actual comparison between the effects of isolated insoluble fiber (cellulose) and cinnamon has been performed in one study, where diabetes-related parameters were monitored for 12 weeks. Cellulose taken as one capsule of 700 mg each morning and another each evening promoted improvement on insulin sensitivity, contrasting the lack of significant effect of capsules containing 500 mg of cinnamon (~243 mg of insoluble fiber) plus 200 mg of cellulose (a total of ~443 mg of insoluble fiber). This result demonstrated that a more pronounced insulin sensitivity was achieved with the ingestion of the capsules containing a higher dose of insoluble fiber (700 mg vs ~443 mg) [153]. As other fibers, cellulose inhibits the activity of carbohydrate-hydrolyzing enzymes [456–458]. The contribution of other macronutrients to the anti-hyperglycemic effect of cinnamon is likely negligible given that the amounts of them are not enough to elicit changes in the postprandial glycemic response, such as protein [471, 472], fat [472, 473], and soluble fiber [474, 475]. The presence of higher amounts of macronutrients in other food that are also rich in insoluble fiber may explain why their lowering-effect of the postprandial glycemic response in T2DM patients is less impressive than cinnamon, e.g., a dose of raw flaxseed providing 3.5 g of insoluble fibre [476]. In conclusion, it is very plausible that the cinnamon’s inhibitory activity is mainly due to its exceptionally high content of insoluble fiber. If this is true, considering that the glycemic response to starch and sucrose is decreased also by cinnamon’s extract [142, 208, 374], which does not contain the bulk of its fiber content, the inhibitory activity should be exerted by some specific insoluble fibers. In fact, some types of insoluble fibers (hemicelluloses) show higher inhibitory activity on α-glucosidase/α-amylase when they are in a bound (feruloylated) form [477], which is the main raw wheat bran phenolic compound, found largely attached (90%) to arabinoxylans [478].

Alternatively, or in combination, there are other molecules or classes of molecules that might contribute to the effect of cinnamon in suppressing the post-meal hyperglycemic spikes in our T2DM patients. These included several volatile molecules and other compounds present in small quantities in the plant [479–482] that are known to inhibit carbohydrate-digesting enzymes, such as cinnamic acid [481, 483–485], cinnamaldehyde [290, 486, 487], procyanidins [139, 481, 488], proanthocyanidin [488–492], eugenol [156, 493–495], safrole [448, 496], coumarin [497–499], benzyl benzoate [482, 500–502], cadinene [503, 504], cinnamyl acetate [94, 482, 505], cinnamyl alcohol [481, 497, 506, 507], kaempferol [508–512], quercetin [343, 509, 513–517], quercetrin [389, 508, 518, 519], protocatechuic acid [520–522], vanillin [521, 523], gallic acid [521, 524, 525], β- and α-pinene [526, 527], chlorogenic acid [480, 528, 529], and benzaldehyde [482, 530–532]. However, the current data do not allow to affirm that the amount of these potential inhibitors in high doses of cinnamon (≥6 g) actually alter digestion and post-meal glycemic responses. Furthermore, some of these bioactive compounds are labile and may be oxidized and lose their functional properties when exposed to air [533], especially with a high surface area to volume ratio of powder preparations.

### Implications

Our findings confirmed the postulates that raw cinnamon cassia inhibits post-meal glucose excursions not only within the harmless euglycemic range (up to ~140–160 mg/dL) but also in the deleterious hyperglycemic levels, reinforcing its potential use as a diabetes treatment strategy [534–536] by inhibiting carbohydrate digestion [344, 367, 400, 410, 438, 484, 490, 537–550]. We chose to use a meal with the healthiest proportion of macronutrients for patients with T2DM [121] in order to have methodological conditions mimicking a real-life context. Therefore, the antihyperglycemic effect observed in the present study could be directly applied in clinical practice. Furthermore, the ingestion of cinnamon dissolved in water did not change markers of taste intensity or palatability of a balanced meal, characteristics that facilitate acceptance of this food intervention and indicate that the consumption of cinnamon is a dietetic management executable by diabetic patients [551]. In addition, cinnamon powder at a dose of 3 g exerted a more pronounced effect in lowering the glucose peak rise than the same dose in capsules, corroborating the findings that capsules decrease the bioavailability of some molecules [299]. Therefore, raw cinnamon encapsulation is not necessary, which increases accessibility to the benefits of this spice and likely improves treatment adherence.

There are several advantages of the use of cinnamon for glycemic control. In addition to its recognized safety [83–85] and widespread use [84, 341, 552, 553], it does not cause flatulence [440, 554–556], diarrhea [381, 557, 558] or dyspepsia [559], as well as common adverse effects of antihyperglycemic drugs [22, 545, 560–562]. Due to its acarbose-like action, raw cinnamon may act as calorie restriction mimetics [536, 545, 563–565], what is in accordance with its effect in promoting loss of visceral fat, waist and weight [88, 131, 133, 135, 331, 342, 534, 566–572]. Moreover, the present findings support that the addition of a moderate dose of raw cinnamon cassia into the main meals is a small and easily applicable strategy that diabetic patients may implement to improve their glucose control, without drastically modifying diet or the food palatability. Still, care should be taken in such implementation, since our findings stem from the specific use of raw cinnamon.

Three decades ago, a study showed that “pressure cooking destroyed the amylase inhibitory activity in sorghum varieties” [169]. In general, heating raw vegetable-derived food by frying, baking and cooking/boiling blunts the inhibitory activity of insoluble fibers on carbohydrate-hydrolyzing enzymes [573]. For example, boiling (98°C) raw pumpkin leaves (Momordica balsamina L.) for only 15 minutes is enough to decrease their inhibitory action on α-amylase and α-glucosidase activities [574]. Similarly, the alpha-amylase inhibitory activity of the extract of commercial cinnamon prepared with boiling or decoction is greatly lower than extracts obtained by four other extraction methods [170, 575]. Part of this effect of heating is due to the decrease in the content of insoluble fiber [165], phenolic molecules [576–578] and volatile compounds [579], and the inactivation/denaturation of bioactive proteins in food [580–583]. Cooking also reduces the content of phytate [584–589], an antidiabetic nutraceutical that inhibits α-amylase that was classified as an antinutrient in the past [590–600]. As a consequence, the antihyperglycemic effect of different foods is suppressed when they are used as ingredient in recipes that involve cooking/boiling or baking [166, 169, 173, 583]. Recent data indicated that cinnamnon extracts prepared with longer exposition to heat cause lower decreases in blood glucose of diabetic animals than extracts made with shorter heating cycles [144]. For these reasons, our findings cannot be readily extrapolated for situations in which cinnamon is not used in its raw form.

In the long term, previous studies on the use of inhibitors of carbohydrate-hydrolyzing enzymes indicate that raw cinnamon might also enhance insulin sensitivity of T2DM patients [265, 398–400]. This outcome greatly depends on key variables and is expected to occur only if: (a) the timing of ingestion is synchronized with the main hyperglycemic meals and not with small snacks [436, 601, 602]; (b) the dose taken matches the amount of carbohydrate sufficiently to attenuate the post-meal glucotoxicity [266, 603]; (c) the ingestion regime decreases the mean amplitude of glycemic excursions (MAGE) [436]; and (d) duration of treatment should be long enough [15, 554, 604]. The lack of control for these factors might explain why some interventional studies promoted glycemic control [361, 568, 570, 605–620] while others did not [153, 379, 380, 555, 571, 621–627]. There are yet other knowledges gaps about the use of cinnamon with the aim to improve diabetes treatment. Long-term intervention trials with well-controlled variables could clarify questions [628, 629] such as the effect of splitting the daily dose of this herb with meals and of the precise time (minutes) prior a meal its intake is more efficient in inhibiting postprandial glucose excursion.

### Limitations

The patients with T2DM evaluated in the present study did not show micro nor macrovascular complications and were not using exogenous insulin; therefore, the results may not be replicated in patients at further advanced stages of T2DM. Only male T2DM patients were included in the protocol to avoid alterations in the glucose metabolism caused by hormonal fluctuations [107] and a postulated abortifacient potential [630], which precludes stating that the effects observed in this work can be reproduced in the female population. Furthermore, we did not quantify the concentration of bioactive compounds in raw cinnamon cassia powder, which can vary greatly between different samples [156, 289, 505, 631, 632] and interferes with the ability to inhibit glucosidase [633]. We did not equalize the fiber content between the control and experimental groups, making plausible that the strong effect of raw cinnamon cassia on postprandial hyperglycemia in T2DM could be exerted mainly by its insoluble fibers and not by any bioactive molecules. Another aspect is that the use of cinnamon powder prevented us from doing a blind experiment that prevented the volunteers from knowing what they were ingesting. The very short-term exposition of our patients to cinnamon did not allow to evaluate whether plant-derived food would promote glycemic control by changing gut microbiota [634, 635]. Finally, our results may not be replicated with milk ingestion, as milk decreases the bioaccessibility of cinnamaldehyde [343] and cinnamon cassia has a low inhibitory effect on lactase [446].

## Conclusion

The present study showed that the intake of a single dose of 3 g of raw cinnamon in the form of powder dissolved in water before a meal containing complex carbohydrates suppressed the glucose spike and the postprandial hyperglycemia in T2DM patients. The intake of raw cinnamon flattened the glycemic curve without changing the shape that is typical of T2DM patients. When the herb was taken in the form of capsules, these anti-hyperglycemic effects were lost or significantly diminished. The cinnamon’s anti-diabetic action confirms its acarbose-like property to inhibit the activities of the carbohydrate-digesting enzymes α-amylases/α-glucosidases, which is in accordance with its exceptionally high content of raw insoluble fiber. Our results reinforce the potential use of raw cinnamon as a diabetes treatment strategy by reducing postprandial hyperglycemia and the consequent and various complications of this condition.

## Supporting information

S1 Protocol Approved trial protocol in original language. Protocol of the study project approved by the ethics committee in original language.
(DOCX)

S2 Protocol Approved trial protocol translated into English. Protocol of the study project approved by the ethics committee translated into English.
(DOCX)

S1 Fig Study flowchart diagram of the participants (following CONSORT). (TIF)

S1 Checklist CONSORT 2010 checklist. (DOCX)
