# Supplementary material for: Suppression of the postprandial hyperglycemia in patients with type 2 diabetes by a raw medicinal herb powder is weakened when consumed in ordinary hard gelatin capsules: A randomized crossover clinical trial
Source: PLoS One. 2024 Oct 9;19(10):e0311501. doi: 10.1371/journal.pone.0311501 (PMC11463819; doi:10.1371/journal.pone.0311501)
Supplement: S1 Protocol — Protocol of the study project approved by the ethics committee in original language. (DOCX) [file pone.0311501.s001.docx]

**Projeto de Pesquisa**

RESPOSTA GLICÊMICA AGUDA APÓS ADIÇÃO DE DIFERENTES ALIMENTOS AO DESJEJUM DE DIABÉTICOS TIPO 2

**Pesquisadora:** Fernanda Duarte Moreira

**Orientador:** Profº Dr. Alexis Welker

**Brasília**

**Janeiro de 2019**

**RESUMO**

**INTRODUÇÃO:** O papel hipoglicemiante de alguns nutrientes tem sido bastante discutido em trabalhos que avaliaram especialmente o efeito a longo prazo. Contudo, avaliar e discutir o efeito glicêmico agudo desses nutrientes é urgente no intuito de estabelecer possíveis manobras nutricionais para ajudar os diabéticos a controlar a hiperglicemia, principalmente a pós-prandial. **OBJETIVO:** Conhecer a resposta glicêmica aguda após a adição de diferentes alimentos ao desjejum de diabéticos tipo 2. **MATERIAL E MÉTODOS:** Trata-se de um ensaio clínico *crossover* com 22 diabéticos tipo 2. Eles realizarão curva glicêmica de 120 minutos após consumo de desjejum acrescido de alimentos com características nutricionais diferentes e variadas dosagens: 3g de canela em pó, 3g de canela em cápsulas; 6 g de canela em pó; 6g de canela em cápsulas; 100g de alface; 100g de tomate; 30ml de azeite de oliva; salada mista com 50g de alface, 50g de tomate e 15 ml de azeite; 150g de abacate; 15g de farelo de trigo; 15g de semente de chia ou 15g de farinha de linhaça dourada, em ordem aleatória. Esse projeto de pesquisa está vinculado ao Programa de Doutorado em Ciências e Tecnologias em Saúde da Faculdade de Ceilândia da Universidade de Brasília. **RESULTADOS ESPERADOS:** Conhecer o impacto glicêmico agudo após a adição de diferentes alimentos em diferentes dosagens à refeição é essencial para indicar tipos e quantidades eficazes para atingir a normoglicemia.

**PALAVRAS-CHAVE:** diabetes mellitus, glicemia, canela, fibras dietéticas, gorduras.

1. **INTRODUÇÃO**

A diabetes mellitus (DM) é um distúrbio caracterizado por hiperglicemia crônica que atinge 425 milhões de pessoas e foi responsável por 4 milhões de mortes no mundo (2017), sendo 44,9% em pessoas com menos de 60 anos de idade (Cho *et al*, 2018). O Brasil é o 4º país com maior número de diabéticos; no período de 2008 a 2010 a diabetes foi responsável por 12% do total de hospitalizações e por 15,4% dos custos hospitalares do Sistema Único de Saúde (SUS) (Costa *et al*, 2017). A adesão terapêutica é o maior desafio para os portadores da doença devido à grande mudança no estilo de vida imposta pelo próprio tratamento (ADA, 2015). No Brasil, a prevalência de diabéticos com controle glicêmico inadequado varia entre 69,3 e 73,1% ( Lima *et al*, 2016; Moreira, 2016) e a hiperglicemia pós-prandial (até 120 minutos após as refeições) pode contribuir com até 70% desse quadro (Riddle *et al*, 2011).

O gerenciamento da hiperglicemia é um grande desafio e muitas estratégias para auxiliar o diabético nessa tarefa têm sido pesquisadas. Vários estudos observaram atividade hipoglicemiante de componentes dietéticos específicos, como fibras, gorduras ou outros nutrientes (Ranasinghe *et al*, 2012; Shen *et al*, 2014; Cao *et al*, 2007; Ley *et al*, 2014; Gopal *et al*, 2017; Boll *et al*, 2016; Perera *et al*, 2012; Vuksan *et al*, 2017). Muitos trabalhos investigaram apenas o efeito hipoglicemiante em médio ou longo prazo, mas a redução glicêmica precisa ser buscada de forma imediata quando o paciente perceber a hiperglicemia e a escolha adequada dos alimentos pode colaborar bastante com esse objetivo (SBD, 2017; Farmer *et al*, 2007).

A canela é um condimento potencial a ser investigado, pois vários ensaios clínicos mostraram que o consumo de 1 a 6g de canela (Cinnamomum cassia), por um período de 40 dias a 4 meses, reduziu a glicêmica em diabéticos tipo 2 (Crawford, 2009; Khan *et al*, 2003; Mang *et al*, 2006; Akilen *et al*, 2010). Porém, o efeito glicêmico agudo em diabéticos após o consumo de diferentes dosagens da canela, em pó ou em cápsulas, não foi investigado.

Sabe-se que o grande consumo de fibras por longos períodos reduz a glicemia em diabéticos (Silva *et al*, 2013), mas o efeito glicêmico agudo após o consumo de diferentes tipos de fibras, em quantidades fisiológicas para uma única refeição (de acordo com as recomendações nutricionais), é necessário para indicar as melhores manobras nutricionais na busca rápida da normoglicemia (SBD, 2017). O consumo de chia, linhaça, farelo de trigo e de vegetais, como o alface, está associado com a redução glicêmica, mas o efeito glicêmico agudo não foi estudado (Vuksan *et al*, 2017; Boll *et al*, 2016; Gopal *et al*, 2017).

Alimentos fontes de gorduras de boa qualidade tambem estão sendo pesquisados. O consumo de abacate mais azeite de oliva, por 4 semanas, promoveu a redução da glicemia em mulheres diabéticas. Entretanto, a resposta glicêmica aguda após o consumo do abacate isolado ou do azeite de oliva puro, ou ainda, do azeite de oliva com salada, não foi pesquisado (Lerman-Garber *et al*, 1994).

Conhecer quais são os alimentos que tem efeito hipoglicemiante, e suas dosagens, é necessário e urgente para auxiliar o diabético na busca do controle glicêmico mais ágil. Vale ressaltar ainda que as abordagens dietéticas tem menor risco de intoxicação ou efeitos colaterais em relação aos medicamentos e podem ser aliadas vantajosas para ajudar esses pacientes na busca constante do controle glicêmico, diminuindo o risco de polifarmácia, interações medicamentosas e efeitos adversos, como a intoxicação.

1. **OBJETIVOS**

**2.1 Geral:** Conhecer a resposta glicêmica aguda após adição de diferentes alimentos ao desjejum de diabéticos tipo 2.

**2.2 Específicos:**

**I)** Medir a resposta glicêmica nos tempos 0/15/30/45/60/90/120 minutos após consumo das refeições testadas que serão:

- Desjejum padrão;

- Desjejum padrão acrescido de 3 g de canela em pó;

- Desjejum padrão acrescido de 3 g de canela em cápsulas;

- Desjejum padrão acrescido de 6 g de canela em pó;

- Desjejum padrão acrescido de 6 g de canela em cápsulas;

- Desjejum padrão acrescido de 100 g de alface;

- Desjejum padrão acrescido de 100 g de tomate;

- Desjejum padrão acrescido de 30 ml de azeite de oliva extra virgem;

- Desjejum padrão acrescido de 115g de salada mista contendo 50 g de alface, 50 g de tomate e 15 ml de azeite de oliva extra virgem;

- Desjejum padrão acrescido de 150 g de abacate;

- Desjejum padrão acrescido de 15 g de farelo de trigo;

- Desjejum padrão acrescido de 15 g de semente de chia e

- Desjejum padrão acrescido de 15 g de farinha de linhaça dourada;

**II)** - Verificar o efeito das refeições testadas sobre a palatabilidade;

**III)** Comparar as respostas glicêmicas tempo a tempo resultantes das refeições avaliadas e

**IV)** - Analisar o comportamento da curva e a da área abaixo da curva glicêmica (nos tempos 0/15/30/45/60/90/120 minutos) após o consumo refeições testadas.

**3. MATERIAL E MÉTODOS**

**3.1 Delineamento experimental:** a pesquisa está delineada como ensaio clínico *crossover*.

3.2 Descrição e seleção da população e amostra: participarão desse estudo 22 sujeitos portadores de diabetes tipo 2 atendidos nos Centros de Saúde do Núcleo Bandeirante, do Riacho Fundo I ou da Candangolândia. Eles serão convidados a participar mediante fixação de cartazes e palestras para divulgação do estudo que acontecerá na UPA do Núcleo Bandeirante. O número da amostra considerou os resultados do estudo de De Carvalho *et al.* (‎2017) e o calculo feito pelo programa *G Power versão 3.1.9.2* (Universidade de Düsseldorf, Alemanha) que indicou a necessidade de pelo menos 19 participantes em cada grupo experimental, ao qual acrescentamos 15% para compensar possíveis perdas. Foi adotado o nível de significância de 5%.

Serão critérios de inclusão: ter diagnóstico de diabetes tipo 2 (American Diabetes Association, 2017); idade entre 30 ≥ e < 60 anos; apresentar consumo regular de desjejum (≥ 100 Kcal ingeridas, dentro de 2 horas após acordar, em ≥ 4 dias da semana); não apresentar alergia aos alimentos testados/usados no estudo; se comprometer a ingerir todos os alimentos do estudo; não ter distúrbios do sono ou fazer uso de medicação para dormir; ter disponibilidade de tempo para participar de todos os encontros no período de até 3 meses e aceitar participar da pesquisa mediante a assinatura o Termo de Consentimento Livre e Esclarecido (TCLE).

Serão excluídos do estudo os sujeitos que usam insulina exógena ou medicamentos para outras patologias que não seja a DM2; tem complicações da diabetes; fumam; possuem distúrbios gastrointestinais ou ritmo intestinal irregular; possuem distúrbios psiquiátricos com comprometimento da compreensão; participam de outros protocolos de pesquisa; se recusam a fornecer ou não sabem informar dados relevantes para a pesquisa e que não comparecerem por 3 vezes aos testes previamente agendados, após terem confirmado a presença no dia anterior à intervenção.

3.3 Entrevista inicial

Os voluntários participarão de entrevista inicial para coleta dos dados: idade, escolaridade, antropometria, tabagismo, tempo de diagnóstico de diabetes, uso, tipo e dosagem de medicamentos, presença de alergia e/ou intolerância alimentar, funcionamento intestinal, número de horas de sono e qualidade do sono auto-referida, hábito de consumo de desjejum e de fibras (**ANEXO I**).

O peso corporal será determinado utilizando balança eletrônica digital e a estatura será aferida utilizando antropômetro vertical. O valor do índice de massa corporal (IMC) será calculado e classificado de acordo com as recomendações da Organização Mundial de Saúde (WHO, 2003). A circunferência da cintura será medida determinando o ponto médio entre a última costela e a crista ilíaca, com uma fita métrica flexível e inelástica (Heyward & Stolarczyk, 2000). Os participantes também passarão por avaliação de composição corporal pelo método de densitometria por dupla emissão de raios-X (DEXA) antes e após o término da intervenção.

Os voluntários serão orientados a seguir prescrição nutricional individualizada, abster-se do álcool e não praticar atividade física nas 24 horas anteriores à participação na pesquisa.

**3.4 Intervenção**

A intervenção acontecerá no período de 01/05 a 30/08/2019.

Os participantes deverão realizar glicemia capilar antes de sair de casa e, caso a glicemia esteja menor que 80mg/dL ou maior que 200mg/dL, não deverão participar da intervenção naquele dia, sendo agendada outra data. Os sujeitos deverão chegar ao local onde será realizada a intervenção (na UPA do Núcleo Bandeirante) no período entre 7h00 e 8h30, e em jejum de 8 a 11 horas. Na noite anterior todos deverão realizar duas refeições padrões (jantar e ceia).

Em ordem aleatória, eles serão orientados a consumir o desjejum padrão, ou desjejum padrão acrescido de um dos alimentos-teste (alimentos que serão testados: 3 g de canela em pó; 3 g de canela em cápsulas; 6 g de canela em pó; 6 g de canela em cápsulas; 100 g de alface; 100 g de tomate; 30 ml de azeite de oliva extra virgem; 115g de salada mista contendo 50 g de alface, 50 g de tomate e 15 ml de azeite de oliva extra virgem; 150 g de abacate; 15 g de farelo de trigo; 15 g de semente de chia ou 15 g de farinha de linhaça dourada), em até 15 minutos. Após o consumo de cada refeição, os participantes preencherão a Escala Analógica Visual (**ANEXO II**) para avaliação da palatabilidade considerando aparência, cheiro, textura, sabor e a intensidade dos sabores (doce, salgado, amargo e azedo) (Flint *et al.*, 2000). A curva glicêmica será monitorada por 120 minutos nos tempos 0, 15, 30, 45, 60, 90 e 120 minutos. A determinação da glicemia capilar será realizada por meio do aparelho Accu-Check Active (Roche Diagnostics) disponibilizado pela pesquisadora e será a opção de escolha para que os resultados glicêmicos sejam conhecidos rapidamente e o paciente não fique exposto ao risco de hipoglicemia não monitorada. A área positiva formada abaixo da curva de resposta glicêmica será calculada pelo método trapezoidal. Aplicação de washout será de no mínimo 3 dias e, no máximo, 10 dias.

A Tabela 1 apresenta a informação nutricional do desjejum padrão que será oferecido para os pacientes. O valor nutricional corresponde à aproximadamente 15% do valor energético total de uma dieta padrão de 2.000kcal (337 Cal), contendo 59,4% de carboidratos, 28,6% de lipídios e 12,0% de proteínas e está de acordo com as Diretrizes da Sociedade Brasileira de Diabetes (2017).

Tabela 1. Informação nutricional do desjejum padrão

| **Alimento** | **Quant** | **CHO (g)** | **LIP (g)** | **PTN (g)** | **Fibras (g)** | **Cal** |
| --- | --- | --- | --- | --- | --- | --- |
| Suco de Fruta | 200ml | 22,00 | 0,00 | 0,00 | 0,90 | 88,00 |
| Torradas salgadas | 40g | 26,67 | 3,20 | 5,47 | 1,47 | 157,36 |
| Queijo processado | 20g | 0,67 | 5,13 | 2,00 | 0,00 | 56,85 |
| Queijo processado *light* | 20g | 0,67 | 2,40 | 2,60 | 0,00 | 34,68 |
| **Total** |  | **50,01** | **10,73** | **10,07** | **2,37** | **336,89** |

Quant: quantidade; CHO: carboidratos; LIP: lipídios; PTN: proteínas; Cal: calorias.

Fonte: Rótulo dos alimentos.

A refeição padrão acrescida dos alimentos a serem testados terão valores similares de carboidratos e proteínas (nutrientes capazes de aumentar a glicemia). Os diabéticos receberão a canela em pó ou o farelo de trigo ou a semente de chia ou a farinha de linhaça adicionados ao suco do desjejum. Em dois experimentos, a canela será oferecida em cápsulas e deverá ser ingerida com o suco. O alface ou o tomate ou o azeite de oliva ou a salada mista ou o abacate serão oferecidos junto com o desjejum e deverão ser consumidos dentro do tempo limite de 15 minutos. Os produtos serão fracionados em balança de alta precisão. Os indivíduos serão orientados a mastigar lentamente e o tempo para ingestão será monitorado e não poderá exceder a 15 minutos com o objetivo de propiciar a absorção dos substratos de forma semelhante entre os indivíduos.

A escolha dos alimentos foi realizada mediante revisão de dados na literatura sobre alimentos com potencial efeito hipoglicemiante e as quantidades das porções foram adotadas considerando a baixa quantidade de carboidrato disponível (que corresponde ao carboidrato total menos a quantidade de fibras) e proteínas, que são os nutrientes capazes de promover elevação glicêmica. A pesquisadora ingeriu todas as refeições a serem testadas para avaliar presença de problemas relacionados à deglutição dos alimentos combinados e das porções adotadas e não houve intercorrências. A Tabela 2 contém as informações nutricionais dos alimentos testados.

**Tabela 2.** Informações nutricionais dos produtos que serão testados

| Alimentos-teste | Quant | Energia  (Cal) | Carb  Total (g) | Gordura  Total (g) | G. Poli  (g) | G. Mono  (g) | G. Sat  (g) | Proteína  (g) | Fibra  Total (g) | Fibra  Solúvel (g) | Fibra  Insolúvel (g) |
| --- | --- | --- | --- | --- | --- | --- | --- | --- | --- | --- | --- |
| Canela em pó | 3g | 10,92 | 2,39 | 0,10 | 0,02 | 0,01 | 0,02 | 0,12 | 1,63 | nd | nd |
| Canela em pó | 6g | 21,84 | 4,79 | 0,19 | 0,03 | 0,03 | 0,04 | 0,24 | 3,26 | nd | nd |
| Alface | 100g | 7,64 | 1,5 | 0,11 | 0,06 | tr | 0,15 | 0,41 | 1,25 | 0,6 | 0,65 |
| Tomate | 100g | 19,44 | 3,82 | 0,17 | 0,08 | 0,03 | 0,03 | 1,04 | 1,03 | 0,25 | 0,78 |
| Azeite de oliva | 30ml | 270 | 0,00 | 30,00 | 2,85 | 22,65 | 4,47 | 0,00 | 0,00 | 0,00 | 0,00 |
| Alface (50g), tomate (50g) e Azeite (15ml) | 115g | 149,8 | 2,66 | 15,14 | 1,495 | 11,34 | 2,325 | 0,725 | 1,14 | 0,425 | 0,715 |
| Abacate | 150g | 125,84 | 8,775 | 9,315 | 1,56 | 4,77 | 2,55 | 1,725 | 6,045 | 2,505 | 3,54 |
| Farelo de trigo | 15g | 53,81 | 9,675 | 0,639 | 0,3315 | 0,096 | 0,0945 | 2,34 | 6,285 | 0,465 | 5,82 |
| Semente de chia | 15g | 84,35 | 6,3 | 5,1585 | 4,0995 | 0,555 | 0,5025 | 3,18 | 6,18 | 0,795 | 5,385 |
| Farinha de linhaça dourada | 15g | 76,8 | 4,5 | 5,2005 | 3,9 | 0,9 | 0,4005 | 3 | 3,9 | 1,2 | 2,7 |

Quant: quantidade; Cal: calorias; CHO: carboidratos; G. Poli: gorduras poliinsaturadas; G. Mono: gorduras monoinsaturadas; G. Sat: gorduras saturadas; tr: traços.

**Fonte:** [Philippi, ST.](https://www.sciencedirect.com/science/article/pii/S2211335518300688#bbb0180) Tabela de composição de alimentos: suporte para decisão nutricional. Editora Manole , Barueri, 2013.

**3.5 Análise Estatística:**

Os dados de caracterização da amostra e palatabilidade das refeições serão analisados pela média ± desvio padrão. Para comparar as médias dos escores de palatabilidade será aplicado o teste de Shapiro-Wilk.

Para examinar os efeitos dos refeições testadas e do tempo sobre a glicemia será feita análise de variância (ANOVA) de duas vias com medidas repetidas com comparações *post hoc* utilizando o ajuste de Bonferroni.

As áreas abaixo da curva (AUC) glicêmica serão calculadas pelo método trapezoidal, considerando a área acima dos valores basais (Tosh, 2013). Serão aplicados os testes de Shapiro-Wilk, Levene e Mauchly para avaliar se os dados apresentam distribuição normal, homocedasticidade e esfericidade, respectivamente. O efeito das refeições testadas sobre a AUC glicêmica ou dos escores de apetite será medido pela ANOVA de uma via com medidas repetidas com comparações *post hoc* de Bonferroni.

Os testes serão realizados com auxílio do software SPSS Statistics versão 21.0 (IBM Corporation), sendo admitido o nível de significância de 5%.

**3.6 Avaliação de riscos:**

Os riscos decorrentes da participação na pesquisa são: ter algum sintoma gastrointestinal ou variações de glicemia, incluindo hipoglicemia. Contudo, a glicemia dos voluntários estará sendo monitorada constantemente e havendo qualquer intercorrência ele direcionado(a) para a Unidade de Pronto Atendimento que funciona no mesmo local (UPA Núcleo Bandeirante).

**3.7 Benefícios:**

O principal benefício dessa pesquisa é conhecer potenciais alimentos com efeito hipoglicemiante agudo que possam auxiliar os diabéticos na busca da normoglicemia.

**3.8 Conflitos de interesses:**

A pesquisadora relata não haver conflito de interesses.

A coleta de dados acontecerá fora do horário de trabalho da servidora Fernanda Duarte Moreira.

**4. REFERÊNCIAS BIBLIOGRÁFICAS:**

Akilen R, Tsiami A, Devendra D, Robinson N. Glycated haemoglobin and blood pressure-lowering effect of cinnamon in multi-ethnic Type 2 diabetic patients in the UK: a randomized, placebo-controlled, double-blind clinical trial. Diabet Med. 2010;27(10):1159–67

American Diabetes Association. Diagnosis and Classification of Diabetes. Diabetes Care. 2015; 38(1):S8-16

American Diabetes Association. Standards of medical care in diabetes– 2017: summary of revisions. Diabetes Care 2017;40:S4–S5

Boll EV, Ekström LM, Courtin CM, Delcour JA, Nilsson AC, Björck IM, Östman EM. [Effects of wheat bran extract rich in arabinoxylan oligosaccharides and resistant starch on overnight glucose tolerance and markers of gut fermentation in healthy young adults.](https://www.ncbi.nlm.nih.gov/pubmed/26169871) Eur J Nutr. 2016 Jun;55(4):1661-70

Cao H, Polansky MM, Anderson RA. Cinnamon extract and polyphenols affect the expression of tristetraprolin, insulin receptor, and glucose transporter 4 in mouse 3 T3-L1 adipocytes. Arch Biochem Biophys. 2007;459(2):214–22

[Cho NH,](https://www.sciencedirect.com/science/article/pii/S0168822718302031#!) [Shaw](https://www.sciencedirect.com/science/article/pii/S0168822718302031#!) JE, [Karuranga S,](https://www.sciencedirect.com/science/article/pii/S0168822718302031#!) [Huang Y,](https://www.sciencedirect.com/science/article/pii/S0168822718302031#!) [da Rocha Fernandes JD,](https://www.sciencedirect.com/science/article/pii/S0168822718302031#!) [Ohlrogge AW,](https://www.sciencedirect.com/science/article/pii/S0168822718302031#!) [Malanda B.](https://www.sciencedirect.com/science/article/pii/S0168822718302031#!) IDF Diabetes Atlas: Global estimates of diabetes prevalence for 2017 and projections for 2045. [Diabetes Research and Clinical Practice](https://www.sciencedirect.com/science/journal/01688227). 2018; 138: 271-281

Costa AF, et al. Carga do diabetes mellitus tipo 2 no Brasil. Cad Saúde Pública. 2017; 33(2):1-14

Crawford P. Effectiveness of cinnamon for lowering hemoglobin A1C in patients with type 2 diabetes: a randomized, controlled trial. J Am Board Fam Med. 2009;22(5):507–12

De Carvalho CM, Paula TP, Viana LV, Machado VMT, Almeida JC. Plasma glucose and insulin responses after consumption of breakfasts with different sources of soluble fiber in type 2 diabetes patients: a randomized crossover clinical trial. Am J Clin Nutr 2017; 106(5): 1238-1245

Farmer A, Wade A, Goyder E, Yudkin P, French D, Craven A et al. Impact of self monitoring of blood glucose in the management of patients with non-insulin treated diabetes: open parallel group randomized trial. BMJ. 2007; 335(7611):132

Flint A, Raben A, Blundell JE, Astrup A. Reproducibility, power and validity of visual analogue scales in assessment of appetite sensations in single test meal studies. Int J Obes Relat Metab Disord 2000;24(1):38-48

Gentilcore D, Chaikomin R, Jones KL, Russo A, Feinle-Bisset C, Wishart JM, Rayner CK, Horowitz M. Effects of Fat on Gastric Emptying of and the Glycemic, Insulin, and Incretin Responses to a Carbohydrate Meal in Type 2 Diabetes, The Journal of Clinical Endocrinology & Metabolism. 2006 Jun; 91(6): 2062–2067

[Gopal SS](https://www.ncbi.nlm.nih.gov/pubmed/?term=Gopal%20SS%5BAuthor%5D&cauthor=true&cauthor_uid=28170007), [Lakshmi MJ](https://www.ncbi.nlm.nih.gov/pubmed/?term=Lakshmi%20MJ%5BAuthor%5D&cauthor=true&cauthor_uid=28170007), [Sharavana G](https://www.ncbi.nlm.nih.gov/pubmed/?term=Sharavana%20G%5BAuthor%5D&cauthor=true&cauthor_uid=28170007), [Sathaiah G](https://www.ncbi.nlm.nih.gov/pubmed/?term=Sathaiah%20G%5BAuthor%5D&cauthor=true&cauthor_uid=28170007), [Sreerama YN](https://www.ncbi.nlm.nih.gov/pubmed/?term=Sreerama%20YN%5BAuthor%5D&cauthor=true&cauthor_uid=28170007), [Baskaran V](https://www.ncbi.nlm.nih.gov/pubmed/?term=Baskaran%20V%5BAuthor%5D&cauthor=true&cauthor_uid=28170007). Lactucaxanthin - a potential anti-diabetic carotenoid from lettuce (Lactuca sativa) inhibits α-amylase and α-glucosidase activity in vitro and in diabetic rats. [Food Funct.](https://www.ncbi.nlm.nih.gov/pubmed/28170007) 2017; 8(3):1124-1131

Heyward VH, Stolarczyk LM. Avaliação da composição corporal aplicada: fundamentos da composição corporal. São Paulo: Manole, 2000

Khan A, Sadafar M, Ali Khan MM, Khattak KN, Anderson RA. Cinnamon improves glucose and lipids of people with type 2 diabetes. Diabetes Care. 2003;26(12):3215–8

[Lerman-Garber I](https://www.ncbi.nlm.nih.gov/pubmed/?term=Lerman-Garber%20I%5BAuthor%5D&cauthor=true&cauthor_uid=8026287), [Ichazo-Cerro S](https://www.ncbi.nlm.nih.gov/pubmed/?term=Ichazo-Cerro%20S%5BAuthor%5D&cauthor=true&cauthor_uid=8026287), [Zamora-González J](https://www.ncbi.nlm.nih.gov/pubmed/?term=Zamora-Gonz%C3%A1lez%20J%5BAuthor%5D&cauthor=true&cauthor_uid=8026287), [Cardoso-Saldaña G](https://www.ncbi.nlm.nih.gov/pubmed/?term=Cardoso-Salda%C3%B1a%20G%5BAuthor%5D&cauthor=true&cauthor_uid=8026287), [Posadas-Romero C](https://www.ncbi.nlm.nih.gov/pubmed/?term=Posadas-Romero%20C%5BAuthor%5D&cauthor=true&cauthor_uid=8026287). Effect of a high-monounsaturated fat diet enriched with avocado in NIDDM patients. [Diabetes Care.](https://www.ncbi.nlm.nih.gov/pubmed/?term=diabetes+control+glycemic+avocado) 1994;17(4):311-5

Ley SH, Hamdy O, Mohan V, Hu FB, 9933 Prevention and management of type 2 diabetes: dietary components and nutritional strategies. Lancet (London, England) 2014;383:1999–2007

Lima RF, Fontbonne A, Carvalho EMF, Montarroyos UR, Barreto MNSC, Cesse EAP. Fatores associados ao controle glicêmico em pessoas com diabetes na Estratégia Saúde da Família em Pernambuco. Revista da Escola de Enfermagem da USP. 2016; 50(6):937-945

Mang B, Wolters M, Schmitt M, Kelb K, Lichtinghagen R, Stichtenoth DO. Effects of a cinnamon extract on plasma glucose, HbA, and serum lipids in diabetes mellitus type 2. Eur J Clin Invest. 2006;36(5):340–4

Moreira SF. Fatores associados ao controle glicêmico inadequado em pacientes com diabetes tipo 2 no Brasil e na Venezuela. 2016. 128 f. Dissertação (Mestrado em Biotecnologia em Saúde e Medicina Investigativa) - Fundação Oswaldo Cruz, Instituto Gonçalo Muniz, Salvador, 2016

Perera PK, Li Y. Functional herbal food ingredients used in type 2 diabetes mellitus. Pharmacogn Rev. 2012;6(11):37–45

[Philippi, ST.](https://www.sciencedirect.com/science/article/pii/S2211335518300688#bbb0180) Tabela de composição de alimentos: suporte para decisão nutricional. Barueri: Editora Manole, 2013

Ranasinghe P, Perera S, Gunatilake M, Abeywardene E, Gunapala N, Premakumara S, Perera K, Lokuhetty D, Katulanda P. Effects of Cinnamomum zeylanicum (Ceylon cinnamon) on blood glucose and lipids in a diabetic and healthy rat model. Pharmacognosy Res. 2012;4(2):73–9

Riddle M, Umpierrez G, DiGenio A, Zhou R, Rosenstock J. Contributions of basal and postprandial hyperglycemia over a wide range of A1C levels before and after treatment intensification in type 2 diabetes. Diabetes Care. 2011; 34:2508–14

Shen YIto Y, Muraki E, Honoso T, Seki T. Cinnamon extract enhances glucose uptake in 3 T3–L1 adipocytes and C2C12 myocytes by inducing LKB1-AMP-activated protein kinase signaling. PLoS One. 2014;9(2)

Silva FM, Kramer CK, de Almeida JC, Steemburgo T, Gross JL, Azevedo MJ. Fiber intake and glycemic control in patients with type 2 diabetes mellitus: a systematic review with meta-analysis of randomized controlled trials. Nutr Rev 2013;71:790–801

Sociedade Brasileira de Diabetes. Diretrizes da Sociedade Brasileira de Diabetes 2017-2018. São Paulo: Editora Clannad, 2017

Tosh SM. Review of human studies investigating the post-prandial blood-glucose lowering ability of oat and barley food products. Eur J ClinNutr 2013;67:310–7

[Vuksan V](https://www.ncbi.nlm.nih.gov/pubmed/?term=Vuksan%20V%5BAuthor%5D&cauthor=true&cauthor_uid=28000689), [Choleva L](https://www.ncbi.nlm.nih.gov/pubmed/?term=Choleva%20L%5BAuthor%5D&cauthor=true&cauthor_uid=28000689), [Jovanovski E](https://www.ncbi.nlm.nih.gov/pubmed/?term=Jovanovski%20E%5BAuthor%5D&cauthor=true&cauthor_uid=28000689), [Jenkins AL](https://www.ncbi.nlm.nih.gov/pubmed/?term=Jenkins%20AL%5BAuthor%5D&cauthor=true&cauthor_uid=28000689), [Au-Yeung F](https://www.ncbi.nlm.nih.gov/pubmed/?term=Au-Yeung%20F%5BAuthor%5D&cauthor=true&cauthor_uid=28000689), [Dias AG](https://www.ncbi.nlm.nih.gov/pubmed/?term=Dias%20AG%5BAuthor%5D&cauthor=true&cauthor_uid=28000689), [Ho HV](https://www.ncbi.nlm.nih.gov/pubmed/?term=Ho%20HV%5BAuthor%5D&cauthor=true&cauthor_uid=28000689), [Zurbau A](https://www.ncbi.nlm.nih.gov/pubmed/?term=Zurbau%20A%5BAuthor%5D&cauthor=true&cauthor_uid=28000689), [Duvnjak L](https://www.ncbi.nlm.nih.gov/pubmed/?term=Duvnjak%20L%5BAuthor%5D&cauthor=true&cauthor_uid=28000689). Comparison of flax (Linum usitatissimum) and Salba-chia (Salvia hispanica L.) seeds on postprandial glycemia and satiety in healthy individuals: a randomized, controlled, crossover study. [Eur J Clin Nutr.](https://www.ncbi.nlm.nih.gov/pubmed/28000689) 2017 Feb;71(2):234-238

World Health Organization. Diet, nutrition and the prevention of chronic diseases. World Health Organ Tech Rep Ser 2003;916:1-149

**ANEXO I**

**QUESTIONÁRIO PARA ENTREVISTA INICIAL**

**I) Dados pessoais**: Data: _____/_____/______

1- Nome: _____________________________________________________________________________

2- Endereço: __________________________________________________________________________

3- Telefone: _________________________________ Celular: __________________________________

4- Data de nascimento: _____/_____/_________ Idade: _____________________________________

5- E-mail: _____________________________________________________________________________

**II) Dados Antropométricos**

6- Peso: ______________________________________________________________________________

7- Altura: _____________________________________________________________________________

8- IMC: _______________________________________________________________________________

9- Circunferência de Cintura: _____________________________________________________________

10- Você fuma:

( ) sim

( ) não

( ) outras: ________________________________________________________________________

11- Usa medicamentos/remédios:

( ) não

( ) sim

Quais/em que horários/posologia: ______________________________________________________

_____________________________________________________________________________________

_____________________________________________________________________________________

_____________________________________________________________________________________

12- Tem alguma alergia ou intolerância alimentar:

( ) não

( ) sim

Se sim, a qual(ais) alimento(s): ____________________________________________________________

_____________________________________________________________________________________

_____________________________________________________________________________________

13- Tem boa tolerância aos seguintes alimentos:

- Néctar de pêssego su fresh ( ) Sim ( ) Não ( ) Não conheço o produto

- Torradas industrializadas ( ) Sim ( ) Não ( ) Não conheço o produto

- Queijo processado ( ) Sim ( ) Não ( ) Não conheço o produto

- Farelo de trigo ( ) Sim ( ) Não ( ) Não conheço o produto

- Farinha de casca de maracujá ( ) Sim ( ) Não ( ) Não conheço o produto

- Pó para gelatina de algas marinhas ( ) Sim ( ) Não ( ) Não conheço o produto

14- Quanto ao funcionamento do seu intestino, é:

( ) regular

( ) irregular

( ) fezes endurecidas

( ) excesso de formação de gases

( ) diarréia

( ) Outros: __________________________________________________________________________

15- Você dorme quantas horas por noite: ___________________________________________________

16- Geralmente, você acorda em qual horário: _______________________________________________

E vai dormir que horas: __________________________________________________________________

17- Você acredita que a qualidade do seu sono é:

( ) Boa ( ) Ruim

18- Você tem distúrbio do sono ou faz uso de medicação para dormir?

( ) Não ( ) Sim Qual (s)? ___________________________________________________________

19- Seu sono é:

( ) Contínuo

( ) Intermitente

Se acorda durante a noite, é por qual motivo e qual o número de vezes: __________________________

_____________________________________________________________________________________

20- Você tem o hábito de consumir café da manhã (refeição com ≥ 100 Kcal ingeridas, dentro de 2 horas após acordar, em ≥ 4 dias da semana):

( ) Sim ( ) Não

**ANEXO II**

**ESCALA DE ANALOGIA VISUAL**

**(***VAS – Visual Analogue Scale***)**

Sabor

Ruim

Ruim

Ruim

Ruim

Boa

Boa

Bom

Boa

Textura

Cheiro

Aparência geral

**Questionário para avaliar a palatabilidade**

Nome voluntário:

___________________________ Nº randomização: _________ Fase:

Data: ________________

Horário: ________

Favor marcar na escala o que melhor reflete a sua resposta para cada uma das questões abaixo:

Quanto

Não estou faminto

Nunca estive

em absoluto

tão faminto

Não estou cheio

em absoluto

Totalmente cheio

Completamente

Não poderia

vazio

comer

mais

nada

Muito mais

Nada mais

Nada

Extremadamente

**Depois de ingerir todos os alimentos da refeição, faça uma avaliação da mesma, considerando os parâmetros apresentados a seguir:**

Sabor azedo

Sabor salgado

Forte

Fraco

Sabor amargo

Forte

Sabor doce

Fraco

Forte

Fraco

Fraco

**Avalie agora a preparação fornecida nesta refeição, quanto à intensidade do sabor apresentado:**
