# Supplementary material for: Suppression of the postprandial hyperglycemia in patients with type 2 diabetes by a raw medicinal herb powder is weakened when consumed in ordinary hard gelatin capsules: A randomized crossover clinical trial
Source: PLoS One. 2024 Oct 9;19(10):e0311501. doi: 10.1371/journal.pone.0311501 (PMC11463819; doi:10.1371/journal.pone.0311501)
Supplement: S2 Protocol — Protocol of the study project approved by the ethics committee translated into English. (DOCX) [file pone.0311501.s002.docx]

**Research project**

ACUTE GLYCEMIC RESPONSE AFTER ADDING DIFFERENT FOODS TO THE BREAKFAST OF PATIENTS WITH TYPE 2 DIABETES

**Researcher:** Fernanda Duarte Moreira

**Advisor:** Prof. Dr. Alexis Welker

**Brasilia**

**January 2019**

**SUMMARY**

**INTRODUCTION:** The hypoglycemic role of some nutrients has been widely discussed in studies that especially evaluated the long-term effect. However, evaluating and discussing the acute glycemic effect of these nutrients is urgent in order to establish possible dietetic management to help patients with diabetes to control hyperglycemia, especially postprandial hyperglycemia. **OBJECTIVE:** To understand the acute glycemic response after the addition of different foods to the breakfast of patients with type 2 diabetes. **MATERIAL AND METHODS:** This is a crossover clinical trial with 22 patients with type 2 diabetes. They will perform a 120-minute glycemic curve after consuming breakfast with different foods showing varied nutritional characteristics and dosages: 3 g of cinnamon powder, 3 g of cinnamon in capsules; 6 g of cinnamon powder; 6 g of cinnamon in capsules; 100 g lettuce; 100 g of tomato; 30 ml olive oil; mixed salad with 50 g of lettuce, 50 g of tomato and 15 ml of olive oil; 150 g of avocado; 15 g of wheat bran; 15 g of chia seeds or 15 g of golden flaxseed flour, in random order. This research project is linked to the Doctoral Program in Health Sciences and Technologies at the Faculty of Ceilândia at the Universidade de Brasília. **EXPECTED RESULTS:** To understand the acute impact on the glycemic response after adding different foods in different dosages to a meal is essential to indicate effective types and quantities to achieve normoglycemia.

**KEYWORDS:** diabetes mellitus, glycemia, cinnamon, dietary fiber, fats.

1. **INTRODUCTION**

Diabetes mellitus (DM) is a disorder characterized by chronic hyperglycemia that affects 425 million people and was responsible for 4 million deaths worldwide (2017), 44.9% of which in people under 60 years of age (Cho *et al* , 2018). Brazil is the 4th country with the highest number of patients with diabetes; in the period from 2008 to 2010, diabetes was responsible for 12% of total hospitalizations and 15.4% of hospital costs in the Unified Health System (SUS) (Costa *et al*, 2017). Therapeutic adherence is the biggest challenge for those with the disease due to the major change in lifestyle imposed by the treatment itself (ADA, 2015). In Brazil, the prevalence of patients with diabetes with inadequate glycemic control varies between 69.3 and 73.1% (Lima *et al*, 2016; Moreira, 2016) and postprandial hyperglycemia (up to 120 minutes after meals) can contribute up to 70% of this picture (Riddle *et al*, 2011).

Managing hyperglycemia is a major challenge and many strategies to assist patients with diabetes in this task have been investigated. Several studies have observed hypoglycemic activity of specific dietary components, such as fiber, fat, or other nutrients (Ranasinghe *et al*, 2012; Shen *et al*, 2014; Dog *et al*, 2007; Ley *et al*, 2014; Gopal *et al*, 2017; Bol *et al*, 2016; Perera *et al*, 2012; Vuksan *et al*, 2017). Many studies have only investigated the hypoglycemic effect in the medium or long-term; however, glycemic reduction needs to be sought immediately when the patient notices hyperglycemia and the appropriate choice of food can greatly contribute to this objective (SBD, 2017; Farmer et *al*, 2007).

Cinnamon is a potential spice to be investigated, as several clinical trials have shown that consuming 1 to 6 g of cinnamon (Cinnamomum cassia) for a period of 40 days to 4 months reduced glycemic levels in patients with type 2 diabetes (Crawford, 2009; Khan *et al*, 2003; Mang *et al*, 2006; Akilen *et al*, 2010). However, the acute effect of cinnamon in the glycemic response in patients with type 2 diabetes after consuming different dosages, in the form of powder or in capsule, has not been investigated.

It is known that high fiber consumption for long periods reduces glycemia in patients with diabetes (Silva *et al*, 2013), but the acute glycemic effect after consuming different types of fiber in physiological amounts for a single meal (according to nutritional recommendations) is necessary to indicate the best dietary modification in the rapid search for normoglycemia (SBD, 2017). The consumption of chia, flaxseed, wheat bran and vegetables, such as lettuce, is associated with decrease in blood glucose level, but the their acute effect on postprandial glycemic response has not been studied (Vuksan *et al* , 2017; Boll *et al*, 2016; Gopal *et al*, 2017).

Food sources of healthy fats are also being investigated. Consumption of avocado plus olive oil for 4 weeks reduced blood glucose levels in women with type 2 diabetes. However, the acute glycemic response after consumption of avocado alone or of olive oil, or of olive oil with salad, has not been studied (Lerman-Garber et al, 1994*)*.

It is necessary and urgent to understand which foods and their dosages show a hypoglycemic effect to help patients with type 2 diabetes in the search for more rapid glycemic control. It is also worth highlighting that dietary approaches have a lower risk of intoxication or side effects compared to medications and can help these patients in the constant search for glycemic control, reducing the risk of polypharmacy, drug interactions and adverse effects, such as intoxication.

1. **OBJECTIVES**

**2.1 General:** To understand the acute glycemic response after adding different foods to the breakfast of patients with type 2 diabetes.

**2.2 Specifics:**

**I)** To measure the glycemic response at times 0/15/30/45/60/90/120 minutes after consumption of the tested meals, which will be:

- Standard breakfast;

- Standard breakfast plus 3 g of cinnamon powder;

- Standard breakfast plus 3 g of cinnamon capsules;

- Standard breakfast plus 6 g of cinnamon powder;

- Standard breakfast plus 6 g of cinnamon capsules;

- Standard breakfast plus 100 g of lettuce;

- Standard breakfast plus 100 g of tomato;

- Standard breakfast plus 30 ml of extra virgin olive oil;

- Standard breakfast plus 115g of mixed salad containing 50 g of lettuce, 50 g of tomato and 15 ml of extra virgin olive oil;

- Standard breakfast plus 150 g of avocado;

- Standard breakfast plus 15 g of wheat bran;

- Standard breakfast plus 15 g of chia seeds and

- Standard breakfast plus 15 g of golden linseed flour;

**II)** – To check the effect of the tested meals on palatability;

**III)** To compare the time-to-time glycemic responses resulting from the evaluated meals and

**IV)** – To analyze the behavior of the curve and the area below the glycemic curve (at times 0/15/30/45/60/90/120 minutes) after consuming tested meals.

**3 . MATERIAL AND METHODS**

**3.1 Experimental design:** the research is designed as a *crossover clinical trial* .

3.2 Description and selection of the population and sample: 22 subjects with type 2 diabetes treated at the Health Centers of Núcleo Bandeirante, Riacho Fundo I or Candangolândia will participate in this study. They will be invited to participate by putting up posters and giving lectures to publicize the study that will take place at the UPA of Núcleo Bandeirante. The sample size was defined considering the results of the study of De Carvalho *et al.* (‎2017) and the calculation was made by the *G Power* program version 3.1.9.2 (University of Düsseldorf, Germany), which indicated the need for at least 19 participants in each experimental group, to which we added 15% to compensate for possible losses. A significance level of 5% was adopted.

The inclusion criteria will be: to have a diagnosis of type 2 diabetes (American Diabetes Association , 2017); age between 30 ≥ and < 60 years; to consume breakfast regularly (≥ 100 kcal ingested, within 2 hours after waking up, on ≥ 4 days of the week); to be not allergic to the foods tested/used in the study; to agree to eat all study foods; to report no sleep disorders or use sleeping medication; to have availability of time to participate in all meetings within a period of up to 3 months and to agree to participate in the research by signing the Free and Informed Consent Form (TCLE).

Subjects that will be excluded from the study are those who: use exogenous insulin or medications for pathologies other than DM2; have complications from diabetes; smoke; have gastrointestinal disorders or irregular intestinal rhythm; have psychiatric disorders with impaired understanding; participate in other research protocols; refuse to provide or are not able to provide relevant data to the research and who do not attend previously scheduled tests for three times after confirming their presence in the day before the intervention.

3.3 Initial interview

Volunteers will participate in an initial interview to collect the following data: age, education, anthropometry, smoking, time since diabetes diagnosis, use, type and dosage of medications, presence of allergy and/or food intolerance, intestinal function, number of hours of sleep and self-reported sleep quality, breakfast and fiber consumption habits (**ANNEX I**).

Body weight will be determined using a digital electronic scale and height will be measured using a vertical anthropometer. The body mass index (BMI) value will be calculated and classified according to the recommendations of the World Health Organization (WHO, 2003) . Waist circumference will be measured by determining the midpoint between the lowest rib and the iliac crest, with a flexible and inelastic measuring tape (Heyward & Stolarczyk, 2000). Participants will also undergo body composition assessment using the double emission X-ray densitometry (DEXA) method before and after the end of the intervention.

Volunteers will be instructed to follow individualized nutritional prescriptions, to abstain from alcohol and physical exercise in the 24 hours prior to participating in the research.

**3.4 Intervention**

The intervention will take place from 05/01 to 08/30/2019.

Participants will measure their capillary blood glucose before leaving home and, if their blood glucose level is lower than 80 mg/dL or higher than 200 mg/dL, they will not participate in the intervention on that day, and another date will be scheduled. Subjects must arrive at the place where the intervention will take place (at the UPA of Núcleo Bandeirante) between 7:00 am and 8:30 am, and fasting for 8 to 11 hours. In the night before, everyone will eat two standard meals (dinner and supper).

In random order, they will be instructed to consume the standard breakfast, or a standard breakfast plus one of the test foods (foods that will be tested: 3 g of cinnamon powder; 3 g of cinnamon in capsules; 6 g of cinnamon powder; 6 g of cinnamon in capsules; 100 g of lettuce; 100 g of tomato; 30 ml of extra virgin olive oil; 115 g of mixed salad containing 50 g of lettuce, 50 g of tomato and 15 ml of extra virgin olive oil; 150 g of avocado; 15 g of wheat bran; 15 g of chia seeds or 15 g of golden flaxseed flour), within 15 minutes. After consuming each meal, participants will complete the Visual Analogue Scale (**ANNEX II**) to assess palatability, considering appearance, smell, texture, flavor and intensity of flavors (sweet, salty, bitter and sour) (Flint *et al.* , 2000). The glycemic curve will be monitored for 120 minutes at times 0, 15, 30, 45, 60, 90 and 120 minutes. Capillary blood glucose determination will be carried out using the Accu-Check Active device (Roche Diagnostics) made available by the researcher and will be the option of choice so that glycemic results are known quickly and the patient is not exposed to the risk of unmonitored hypoglycemia. The positive area formed below the glycemic response curve will be calculated using the trapezoidal method. Washout application will be for a minimum of 3 days and a maximum of 10 days.

Table 1 presents the nutritional information of the standard breakfast that will be offered to patients. The nutritional value corresponds to approximately 15% of the total energy value of a standard diet of 2,000 kcal, containing 59.4% carbohydrates, 28.6% lipids and 12.0% proteins and is in accordance with the Brazilian Diabetes Society Guidelines (2017).

Table 1. Nutritional information for the standard breakfast

| **Food** | **Quantity** | **CHO (g)** | **LIP (g)** | **PTN (g)** | **Fibers (g)** | **Lime** |
| --- | --- | --- | --- | --- | --- | --- |
| Fruit juice | 200ml | 22.00 | 0.00 | 0.00 | 0.90 | 88.00 |
| Savory toast | 40g | 26.67 | 3.20 | 5.47 | 1.47 | 157.36 |
| Processed cheese | 20g | 0.67 | 5.13 | 2.00 | 0.00 | 56.85 |
| *Light* processed cheese | 20g | 0.67 | 2.40 | 2.60 | 0.00 | 34.68 |
| **Total** |  | **50.01** | **10.73** | **10.07** | **2.37** | **336.89** |

Quantity: quantity; CHO: carbohydrates; LIP: lipids; PTN: proteins; Cal: calories.

Source: Food label.

The standard meal plus the foods to be tested will have similar amounts of carbohydrates and proteins (nutrients capable of increasing blood glucose). Patients with diabetes will have cinnamon powder or wheat bran or chia seed or flaxseed flour added to their breakfast juice. In two experiments, cinnamon will be offered in capsules and must be ingested with the juice. Lettuce or tomato or olive oil or mixed salad or avocado will be offered with breakfast and must be consumed within the 15-minute time limit. The products will be divided on a high-precision scale. Individuals will be instructed to chew slowly and the time for ingestion will be monitored and cannot exceed 15 minutes with the aim of promoting the absorption of substrates in a similar way among individuals.

The choice of foods was made by reviewing data in the literature on foods with potential hypoglycemic effects and the portion sizes were adopted considering the low amount of carbohydrate available (which corresponds to total carbohydrate minus the amount of fiber) and proteins, which are the nutrients capable of promoting glycemic elevation. The researcher ate all the meals to be tested to assess the presence of problems related to swallowing the combined foods and portions adopted, and there were no complications. Table 2 contains the nutritional information for the foods tested.

**Table 2.** Nutritional information of the products that will be tested

| Test foods | Quantity | Energy  (Lime) | Carbs  Total (g) | Fat  Total (g) | G. Poli  (g) | G. Mono  (g) | G. Sat  (g) | Protein  (g) | Fiber  Total (g) | Fiber  Soluble (g) | Fiber  Insoluble (g) |
| --- | --- | --- | --- | --- | --- | --- | --- | --- | --- | --- | --- |
| Cinnamon powder | 3g | 10.92 | 2.39 | 0.10 | 0.02 | 0.01 | 0.02 | 0.12 | 1.63 | na | na |
| Cinnamon powder | 6g | 21.84 | 4.79 | 0.19 | 0.03 | 0.03 | 0.04 | 0.24 | 3.26 | na | na |
| Lettuce | 100g | 7.64 | 1.5 | 0.11 | 0.06 | tr | 0.15 | 0.41 | 1.25 | 0.6 | 0.65 |
| Tomato | 100g | 19.44 | 3.82 | 0.17 | 0.08 | 0.03 | 0.03 | 1.04 | 1.03 | 0.25 | 0.78 |
| Olive oil | 30ml | 270 | 0.00 | 30.00 | 2.85 | 22.65 | 4.47 | 0.00 | 0.00 | 0.00 | 0.00 |
| Lettuce (50g), tomato (50g) and Olive Oil (15ml) | 115g | 149.8 | 2.66 | 15.14 | 1,495 | 11.34 | 2,325 | 0.725 | 1.14 | 0.425 | 0.715 |
| Avocado | 150g | 125.84 | 8,775 | 9,315 | 1.56 | 4.77 | 2.55 | 1,725 | 6,045 | 2,505 | 3.54 |
| Wheat bran | 15g | 53.81 | 9,675 | 0.639 | 0.3315 | 0.096 | 0.0945 | 2.34 | 6,285 | 0.465 | 5.82 |
| chia seed | 15g | 84.35 | 6.3 | 5.1585 | 4.0995 | 0.555 | 0.5025 | 3.18 | 6.18 | 0.795 | 5,385 |
| Golden flaxseed flour | 15g | 76.8 | 4.5 | 5.2005 | 3.9 | 0.9 | 0.4005 | 3 | 3.9 | 1.2 | 2.7 |

Quantity: quantity; Cal: calories; CHO: carbohydrates; G. Poly: polyunsaturated fats ; G. Mono: monounsaturated fats; G. Sat: saturated fats; tr : dashes.

**Source:** [Philippi , ST.](https://www.sciencedirect.com/science/article/pii/S2211335518300688#bbb0180) Food composition table: support for nutritional decision-making . Editora Manole, Barueri, 2013.

**3.5 Statistical Analysis:**

Sample characterization data and meal palatability will be analyzed using the mean ± standard deviation. To compare the average palatability scores, the Shapiro- Wilk test will be applied.

To examine the effects of the tested meals and time on blood glucose, two-way analysis of variance (ANOVA) with repeated measures will be performed with *post hoc* comparisons using the Bonferroni adjustment.

The areas under the glycemic curve (AUC) will be calculated using the trapezoidal method, considering the area above baseline values (Tosh, 2013). The Shapiro-Wilk, Levene and Mauchly tests will be applied to assess whether the data present normal distribution, homoscedasticity and sphericity, respectively. The effect of the tested meals on glycemic AUC or appetite scores will be measured by one-way ANOVA with repeated measures with Bonferroni *post hoc comparisons* .

The tests will be carried out using the SPSS Statistics software version 21.0 (IBM Corporation) with a significance level of 5% being accepted.

**3.6 Risk assessment:**

The risks arising from participating in the research are: to show any gastrointestinal symptoms or variations in blood glucose, including hypoglycemia. However, the volunteers' blood glucose will be constantly monitored and, if there is any complication, they will be directed to the Emergency Care Unit that operates in the same location (UPA Núcleo Bandeirante).

**3.7 Benefits:**

The main benefit of this research is to understand potential foods with an acute hypoglycemic effect that can help patients with diabetes in the search for normoglycemia.

**3.8 Conflicts of interest:**

The researcher reports that there is no conflict of interest.

Data collection will take place outside Fernanda Duarte Moreira's working hours.

**4. BIBLIOGRAPHIC REFERENCES:**

Akilen R, Tsiami A, Devendra D, Robinson N. Glycated haemoglobin and blood pressure-lowering effect of cinnamon in multi-ethnic Type 2 diabetic patients in the UK: a randomized , placebo- controlled , double-blind clinical trial . Diabet Med. 2010;27(10):1159–67

American Diabetes Association . Diagnosis and Classification of Diabetes. Diabetes Care . 2015; 38(1):S8-16

American Diabetes Association . Standards of medical care in diabetes – 2017: summary of revisions . Diabetes Care 2017;40:S4–S5

Boll EV, Ekström LM, Courtin CM, Delcour JA, Nilsson AC, Björck IM, Östman EM. [Effects of wheat white extract rich in arabinoxylan oligosaccharides and resistant starch on overnight glucose tolerance and markers of gut fermentation in healthy young adults .](https://www.ncbi.nlm.nih.gov/pubmed/26169871) Eur J Nutr. 2016 Jun;55(4):1661-70

Cao H, Polansky MM, Anderson RA. Cinnamon extract and polyphenols affect the expression of Tristetraprolin , insulin receptor, and glucose transporter 4 in mouse 3 T3-L1 adipocytes . Arch Biochem Biophys . 2007;459(2):214–22

[Cho NH,](https://www.sciencedirect.com/science/article/pii/S0168822718302031#!) [Shaw](https://www.sciencedirect.com/science/article/pii/S0168822718302031#!) JE, [Karuranga S,](https://www.sciencedirect.com/science/article/pii/S0168822718302031#!) [Huang Y,](https://www.sciencedirect.com/science/article/pii/S0168822718302031#!) [da Rocha Fernandes JD,](https://www.sciencedirect.com/science/article/pii/S0168822718302031#!) [Ohlrogge AW,](https://www.sciencedirect.com/science/article/pii/S0168822718302031#!) [Malanda B.](https://www.sciencedirect.com/science/article/pii/S0168822718302031#!) IDF Diabetes Atlas: Global estimates of diabetes prevalence for 2017 and projections for 2045. [Diabetes Research and Clinical Practice](https://www.sciencedirect.com/science/journal/01688227) . 2018; 138: 271-281

Costa AF, et al. Burden of type 2 diabetes mellitus in Brazil. Cad Public Health. 2017; 33(2):1-14

Crawford P. Effectiveness of cinnamon for lowering hemoglobin A1C in patients with type 2 diabetes: a randomized , controlled trial . J Am Board Fam Med. 2009;22(5):507–12

De Carvalho CM, Paula TP, Viana LV, Machado VMT, Almeida JC. Plasma glucose and insulin responses after consumption of breakfasts with different sources of soluble fiber in type 2 diabetes patients : a randomized crossover clinical trial . Am J Clin Nutr 2017; 106(5): 1238-1245

Farmer A, Wade A, Goyder E, Yudkin P, French D, Craven A et al. Impact of self monitoring of blood glucose in the management of patients with non- insulin treated diabetes: open parallel group randomized trial . BMJ. 2007; 335(7611):132

Flint A, Raben A, Blundell JE, Astrup A. Reproducibility, power and validity of visual analogue scales in assessment of appetite sensations in single test meal studies. Int J Obes Report Metab Disord 2000; 24(1):38-48

Gentilcore D, Chaikomin R, Jones KL, Russo A, Feinle-Bisset C, Wishart JM, Rayner CK, Horowitz M. Effects of Fat on Gastric Emptying of and the Glycemic , Insulin , and Incretin Responses to a Carbohydrate Meal in Type 2 Diabetes, The Journal of Clinical Endocrinology & Metabolism . 2006 Jun ; 91(6): 2062–2067

[Gopal SS](https://www.ncbi.nlm.nih.gov/pubmed/?term=Gopal%20SS%5BAuthor%5D&cauthor=true&cauthor_uid=28170007) , [Lakshmi MJ](https://www.ncbi.nlm.nih.gov/pubmed/?term=Lakshmi%20MJ%5BAuthor%5D&cauthor=true&cauthor_uid=28170007) , [Sharavana G](https://www.ncbi.nlm.nih.gov/pubmed/?term=Sharavana%20G%5BAuthor%5D&cauthor=true&cauthor_uid=28170007) , [Sathaiah G](https://www.ncbi.nlm.nih.gov/pubmed/?term=Sathaiah%20G%5BAuthor%5D&cauthor=true&cauthor_uid=28170007) , [Sreerama YN](https://www.ncbi.nlm.nih.gov/pubmed/?term=Sreerama%20YN%5BAuthor%5D&cauthor=true&cauthor_uid=28170007) , [Baskaran V](https://www.ncbi.nlm.nih.gov/pubmed/?term=Baskaran%20V%5BAuthor%5D&cauthor=true&cauthor_uid=28170007) . Lactucaxanthin - a potential anti-diabetic carotenoid from lettuce ( Lactuca sativa ) inhibits α- amylase and α- glucosidase activity in vitro and in diabetics rats . [Food Function .](https://www.ncbi.nlm.nih.gov/pubmed/28170007) 2017; 8(3):1124-1131

Heyward VH, Stolarczyk LM. Assessment of applied body composition: fundamentals of body composition. São Paulo: Manole, 2000

Khan A, Sadafar M, Ali Khan MM, Khattak KN, Anderson RA. Cinnamon improves glucose and lipids of people with type 2 diabetes. Diabetes Care . 2003;26(12):3215–8

[Lerman-Garber I](https://www.ncbi.nlm.nih.gov/pubmed/?term=Lerman-Garber%20I%5BAuthor%5D&cauthor=true&cauthor_uid=8026287) , [Ichazo -Cerro S](https://www.ncbi.nlm.nih.gov/pubmed/?term=Ichazo-Cerro%20S%5BAuthor%5D&cauthor=true&cauthor_uid=8026287) , [Zamora-González J](https://www.ncbi.nlm.nih.gov/pubmed/?term=Zamora-Gonz%C3%A1lez%20J%5BAuthor%5D&cauthor=true&cauthor_uid=8026287) , [Cardoso- Saldaña G](https://www.ncbi.nlm.nih.gov/pubmed/?term=Cardoso-Salda%C3%B1a%20G%5BAuthor%5D&cauthor=true&cauthor_uid=8026287) , [Posadas-Romero](https://www.ncbi.nlm.nih.gov/pubmed/?term=Posadas-Romero%20C%5BAuthor%5D&cauthor=true&cauthor_uid=8026287) C. Effect of a high- monounsaturated fat diet enriched with avocado in NIDDM patients . [Diabetes Care .](https://www.ncbi.nlm.nih.gov/pubmed/?term=diabetes+control+glycemic+avocado) 1994;17(4):311-5

Ley SH, Hamdy O, Mohan V, Hu FB, 9933 Prevention and management of type 2 diabetes: dietary components and nutritional strategies . Lancet (London, England ) 2014;383:1999–2007

Lima RF, Fontbonne A, Carvalho EMF, Montarroyos UR, Barreto MNSC, Cesse EAP. Factors associated with glycemic control in people with diabetes in the Family Health Strategy in Pernambuco. Magazine of the USP School of Nursing. 2016; 50 (6):937-945

Mang B, Wolters M, Schmitt M, Kelb K, Lichtinghagen R, Stichtenoth DO. Effects of a cinnamon extract on plasma glucose, HbA , and be a lipids in type 2 diabetes mellitus . Eur J Clin Invest. 2006;36(5):340–4

Moreira SF. Factors associated with inadequate glycemic control in patients with type 2 diabetes in Brazil and Venezuela. 2016. 128 f. Dissertation (Masters in Biotechnology in Health and Investigative Medicine) - Fundação Oswaldo Cruz, Instituto Gonçalo Muniz, Salvador, 2016

Perera PK, Li Y. Functional herbal food ingredients used in type 2 diabetes mellitus. Pharmacogn Rev. 2012;6(11):37–45

[Philippi , ST.](https://www.sciencedirect.com/science/article/pii/S2211335518300688#bbb0180) Food composition table: support for nutritional decision-making . Barueri: Editora Manole, 2013

Ranasinghe P, Perera S, Gunatilake M, Abeywardene E, Gunapala N, Premakumara S, Perera K, Lokuhetty D, Katulanda P. Effects of Cinnamomum zeylanicum ( Ceylon cinnamon ) on blood glucose and lipids in a diabetic and healthy rat model . Pharmacognosy Res. 2012;4(2):73–9

Riddle M, Umpierrez G, DiGenio A, Zhou R, Rosenstock J. Contributions of basal and postprandial hyperglycemia over a wide range of A1C levels before and after treatment intensification in type 2 diabetes. Diabetes Care . 2011; 34:2508–14

Shen YIto Y, Muraki E, Honoso T, Seki T. Cinnamon extract enhances glucose uptake in 3 T3–L1 adipocytes and C2C12 myocytes by inducing LKB1-AMP-activated protein kinase signaling . PLoS One . 2014; 9 (2)

Silva FM, Kramer CK, de Almeida JC, Steemburgo T, Gross JL, Azevedo MJ. fiber intake and glycemic control in patients with type 2 diabetes mellitus: a systematic review with meta- analysis of randomized controlled trials . Nurture Rev 2013;71:790–801

Brazilian Diabetes Society. Brazilian Diabetes Society Guidelines 2017-2018. São Paulo: Editora Clannad , 2017

Tosh SM. Review of human studies investigating the postprandial blood -glucose lowering ability of oat and barley food products . Eur J ClinNutr 2013;67:310–7

[Vuksan V](https://www.ncbi.nlm.nih.gov/pubmed/?term=Vuksan%20V%5BAuthor%5D&cauthor=true&cauthor_uid=28000689) , [Choleva L](https://www.ncbi.nlm.nih.gov/pubmed/?term=Choleva%20L%5BAuthor%5D&cauthor=true&cauthor_uid=28000689) , [Jovanovski E](https://www.ncbi.nlm.nih.gov/pubmed/?term=Jovanovski%20E%5BAuthor%5D&cauthor=true&cauthor_uid=28000689) , Jenkins [AL](https://www.ncbi.nlm.nih.gov/pubmed/?term=Jenkins%20AL%5BAuthor%5D&cauthor=true&cauthor_uid=28000689) , [Au -Yeung F](https://www.ncbi.nlm.nih.gov/pubmed/?term=Au-Yeung%20F%5BAuthor%5D&cauthor=true&cauthor_uid=28000689) , [Dias AG](https://www.ncbi.nlm.nih.gov/pubmed/?term=Dias%20AG%5BAuthor%5D&cauthor=true&cauthor_uid=28000689) , [Ho HV](https://www.ncbi.nlm.nih.gov/pubmed/?term=Ho%20HV%5BAuthor%5D&cauthor=true&cauthor_uid=28000689) , [Zurbau A](https://www.ncbi.nlm.nih.gov/pubmed/?term=Zurbau%20A%5BAuthor%5D&cauthor=true&cauthor_uid=28000689) , [Duvnjak L.](https://www.ncbi.nlm.nih.gov/pubmed/?term=Duvnjak%20L%5BAuthor%5D&cauthor=true&cauthor_uid=28000689) Comparison of flax ( Linum usitatissimum ) and Salba -chia ( Salvia hispanica L.) seeds on postprandial glycemia and satiety in healthy individuals : a randomized , controlled , crossover study . [Eur J Clin Nutr.](https://www.ncbi.nlm.nih.gov/pubmed/28000689) 2017 Feb;71(2):234-238

World Health Organization . Diet, nutrition and the prevention of chronic diseases . World Health Organ Tech Rep Ser 2003;916:1-149

**ANNEX I**

**QUESTIONNAIRE FOR INITIAL INTERVIEW**

**I) Personal data** : Date: _____/_____/______

1- Name: ________________________________________________________________________________

2- Address: _____________________________________________________________________________

3- Telephone: _________________________________ Cell phone: __________________________________

4- Date of birth: _____/_____/_________ Age: _____________________________________

5- Email: ________________________________________________________________________________

**II) Anthropometric Data**

6- Weight: ______________________________________________________________________________

7- Height: ________________________________________________________________________________

8- BMI: ______________________________________________________________________________

9- Waist Circumference: _____________________________________________________________

10- You smoke:

( ) Yes

( ) no

( ) others: ________________________________________________________________________

11- Uses medicines/medicines:

( ) no

( ) Yes

Which/at what times/dosage: ______________________________________________________

_____________________________________________________________________________________

_____________________________________________________________________________________

_____________________________________________________________________________________

12- Do you have any allergies or food intolerances:

( ) no

( ) Yes

If yes, which food(s): ___________________________________________________________

_____________________________________________________________________________________

_____________________________________________________________________________________

13- You have good tolerance to the following foods:

- Peach nectar su fresh ( ) Yes ( ) No ( ) I don't know the product

- Industrialized toast ( ) Yes ( ) No ( ) I don't know the product

- Processed cheese ( ) Yes ( ) No ( ) I don’t know the product

- Wheat bran ( ) Yes ( ) No ( ) I don’t know the product

- Passion fruit peel flour ( ) Yes ( ) No ( ) I don't know the product

- Seaweed gelatin powder ( ) Yes ( ) No ( ) I don't know the product

14- Regarding the functioning of your intestine, it is:

( ) regular

( ) irregular

( ) hard stools

( ) excess gas formation

( ) diarrhea

( ) Others: __________________________________________________________________________

15- How many hours do you sleep per night: ______________________________________________________

16- What time do you generally wake up at: _______________________________________________

And what time will you go to sleep: _____________________________________________________________________

17- Do you believe that the quality of your sleep is:

( ) Good ( ) Bad

18- Do you have a sleep disorder or use sleeping medication?

( ) No ( ) Yes Which one(s)? _____________________________________ ___________________

19- Your sleep is:

( ) Continuous

( ) Intermittent

If you wake up during the night, for what reason and how many times: __________________________

_____________________________________________________________________________________

20- You have the habit of consuming breakfast (meal with ≥ 100 Kcal ingested, within 2 hours of waking up, on ≥ 4 days of the week ):

( ) Yes No

**ANNEX II**

**VISUAL ANALOGY SCALE**

**(** *VAS – Visual Analogue Scale* **)**

Smell

Pleasantness

Bad

Bad

Bad

Bad

Good

Good

Good

Good

Texture

Visual appeal

**Questionnaire to evaluate palatability**

Volunteer name:

___________________________ Nº randomização: _________ Fase:

Date: ________________

Time: ________

Favor marcar na escala o que melhor reflete a sua resposta para cada uma das questões abaixo:

Não estou faminto

Nunca estive

em absoluto

tão faminto

Não estou cheio

em absoluto

Totalmente cheio

Completamente

Não poderia

vazio

comer

mais

nada

Muito mais

Nada mais

Nada

Extremadamente

**After eating all the food in the meal, evaluate it, considering the parameters presented below:**

Weak

Strong

Sourness

Saltiness

Strong

Weak

Saltiness

Strong

Sweetness

Weak

Strong

Weak

**Now evaluate the preparation provided in this meal, regarding the intensity of the flavor presented:**
