# Supplementary figures and images for: Suppression of the postprandial hyperglycemia in patients with type 2 diabetes by a raw medicinal herb powder is weakened when consumed in ordinary hard gelatin capsules: A randomized crossover clinical trial
Source: PLoS One. 2024 Oct 9;19(10):e0311501. doi: 10.1371/journal.pone.0311501 (PMC11463819; doi:10.1371/journal.pone.0311501)

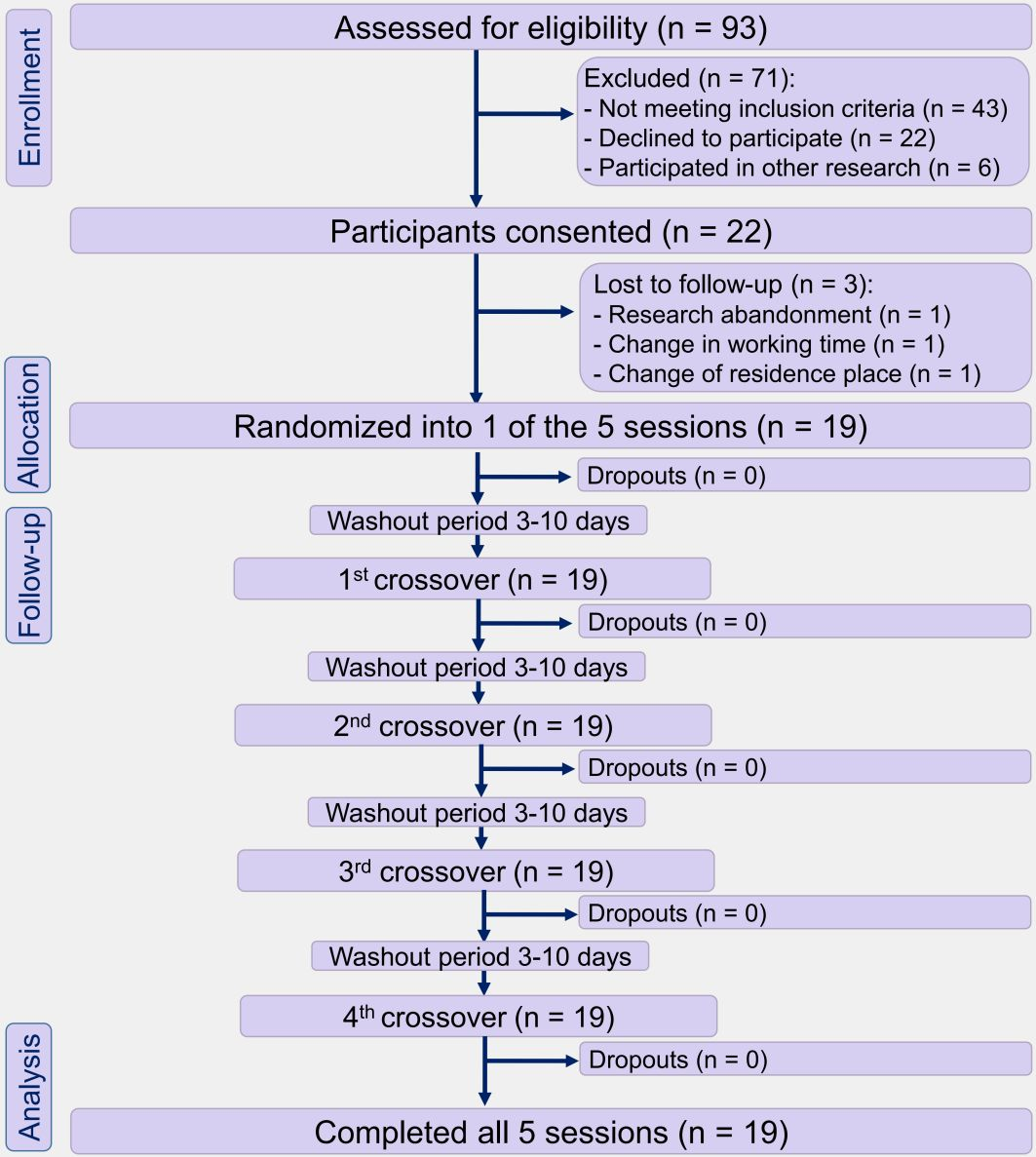

Supplement: S1 Fig — (TIF) [file pone.0311501.s003.tif]
